# Supplementary material for: Five dominant amino acid substitution signatures shape tumour immunity
Source: Mol Syst Biol. 2026 Jan 28;22(5):766–86. doi: 10.1038/s44320-026-00193-x (PMC13144524; doi:10.1038/s44320-026-00193-x)
Supplement: Supplementary file 1 — Appendix [file 44320_2026_193_MOESM1_ESM.pdf]

Appendix to *Five dominant amino acid substitution signatures shape tumour immunity*

Szilvia Juhász, Benjamin Tamás Papp, Anna Tácia Fülöp, Zoltán Farkas, Dávid Kókai, Dóra Alexandra Gyémánt, Franciska Tóth, Zsófia Nacsa, Dóra Spekhardt, Balázs Koncz, Péter Burkovics, Csaba Pál & Máté Manczinger

Table of Contents

Supplementary text: **2**
Appendix Figure S1. .... **3**
Appendix Figure S2. .... **4**
Appendix Figure S3. .... **5**
Appendix Figure S4. .... **6**
Appendix Figure S5. .... **7**
Appendix Figure S6. .... **8**
Appendix Figure S7. .... **9**
Appendix Figure S8. .... **10**
Appendix Figure S9. .... **11**
Appendix Figure S10. .... **13**
Appendix Figure S11. .... **14**
Appendix Figure S12. .... **15**
Appendix Figure S13. .... **16**
Appendix Figure S14. .... **17**
Appendix Figure S15. .... **18**
Appendix Figure S16. .... **19**
Appendix Figure S17. .... **20**
Appendix Table S1. .... **21**
Appendix Table S2. .... **22**
Appendix Table S3. .... **23**
Appendix Table S4. .... **24**
Appendix Table S5. .... **25**
Appendix Table S6. .... **26**
Appendix Table S7. .... **27**

Supplementary text:

In this analysis, we aimed to validate the influence of amino acid substitution signatures (AASs) on neopeptide repertoire presented by human leukocyte antigen (HLA) molecules on cancer cell surfaces. To this end, we analyzed previously published systematic immunopeptidomics datasets profiling HLA-I–bound neopeptides from 17 cancer samples with matched genomic data describing their somatic mutations (See Appendix Table S7).

As throughout the paper, the analysis was restricted to missense mutations. We first computed the cosine similarity between the relative frequency of observed amino acid substitutions in each sample and each AAS (Appendix Figure S16). For each sample, all possible amino acid substitutions (n = 166) were then classified as either detected or not detected in HLA-I–bound neopeptides. For each substitution, we calculated an association score to the five AASs by summing their association strengths, weighted by the cosine similarity between the given sample and each AAS (see Methods). Using substitution detection status across samples (detected vs. undetected, n = 17 × 166) as the response variable and the weighted association scores as predictors, we constructed receiver operating characteristic (ROC) curves. This analysis yielded an area under the ROC curve (AUC) of 0.79 (Appendix Figure S17). As a control, we repeated the analysis 100 times after randomizing amino acid substitution labels, obtaining ROC AUC values ranging from 0.445 to 0.612 (mean = 0.535, SD = 0.033). Together, these results indicate that amino acid substitution context, as captured by AASs, shapes the cancer immunopeptidome.

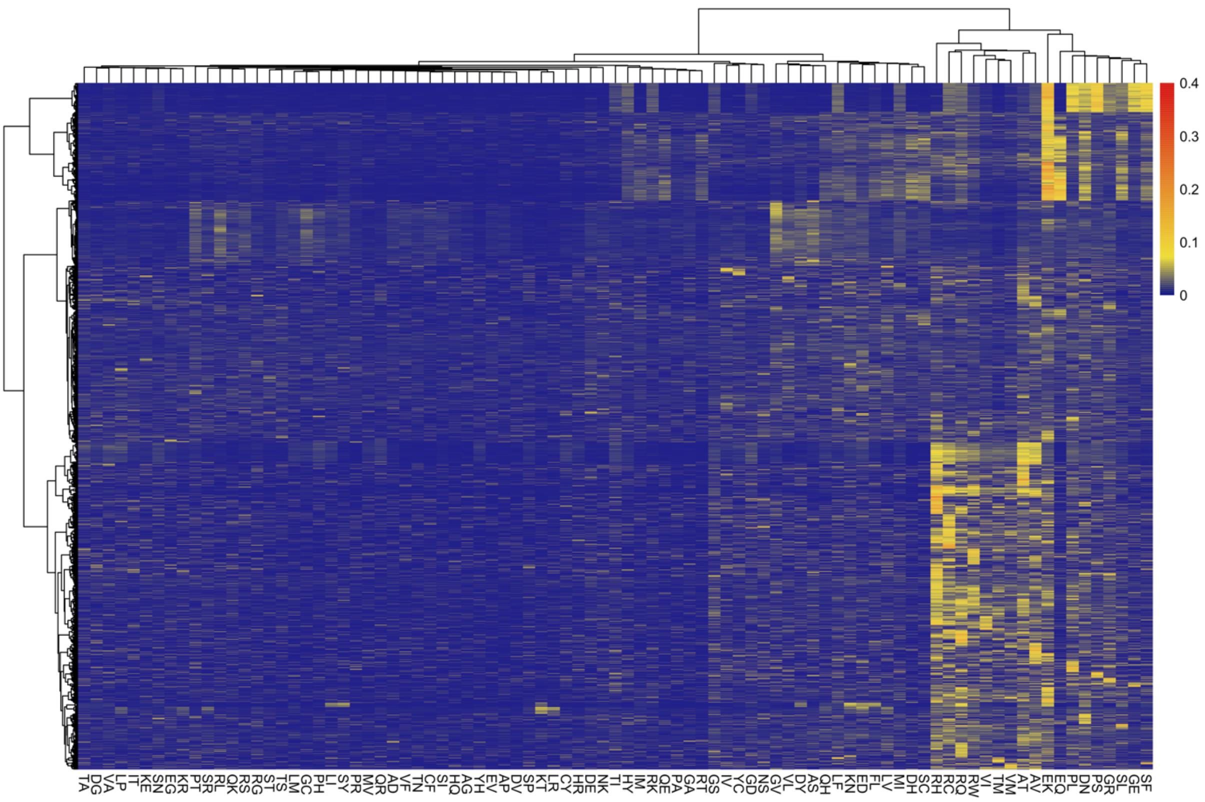

Appendix Figure S1.

The frequency of amino acid substitutions varies widely between samples. The heatmap indicates the relative fraction of amino acid substitutions in each sample in the TCGA database color-coded (n = 9374 samples). Only substitutions with a variance of at least 10^{-4} are indicated. Every row corresponds to one sample. The rows (samples) and columns (substitutions) are clustered using the Euclidean distance and the ward.D2 hierarchical clustering method. The hierarchy trees are shown on the left side and on the top of the figure. Each amino acid substitution is denoted by the single-letter codes of the original and the mutated amino acids.

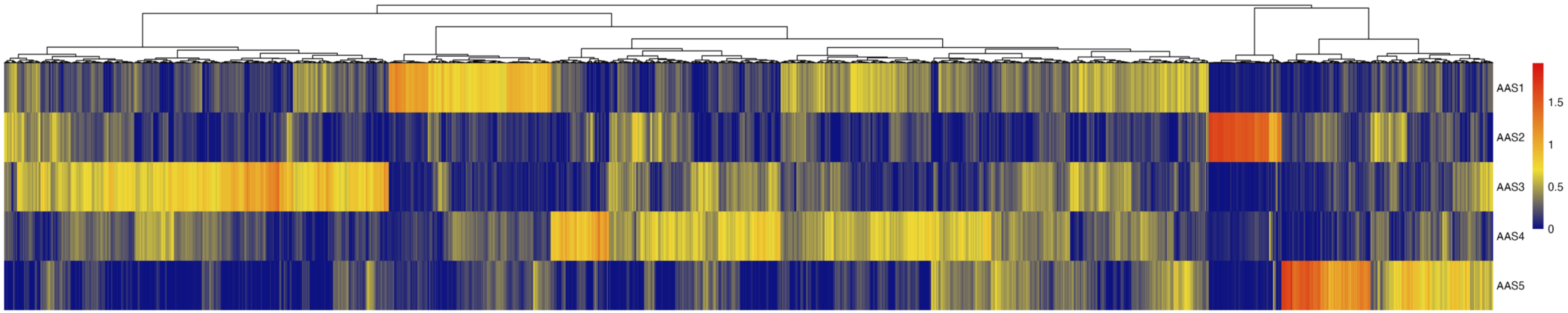

Appendix Figure S2.

The prevalence of AASs in cancer samples. The prevalence of each AAS is indicated in each tumour sample color-coded (n = 9384 samples). The extracted basis components of the original amino acid substitution matrix are treated as a proxy of AAS prevalence in samples. Every column corresponds to a tumour sample. The columns (samples) are clustered using the Euclidean distance and the ward.D2 hierarchical clustering method. The hierarchy tree is shown on the top of the figure.

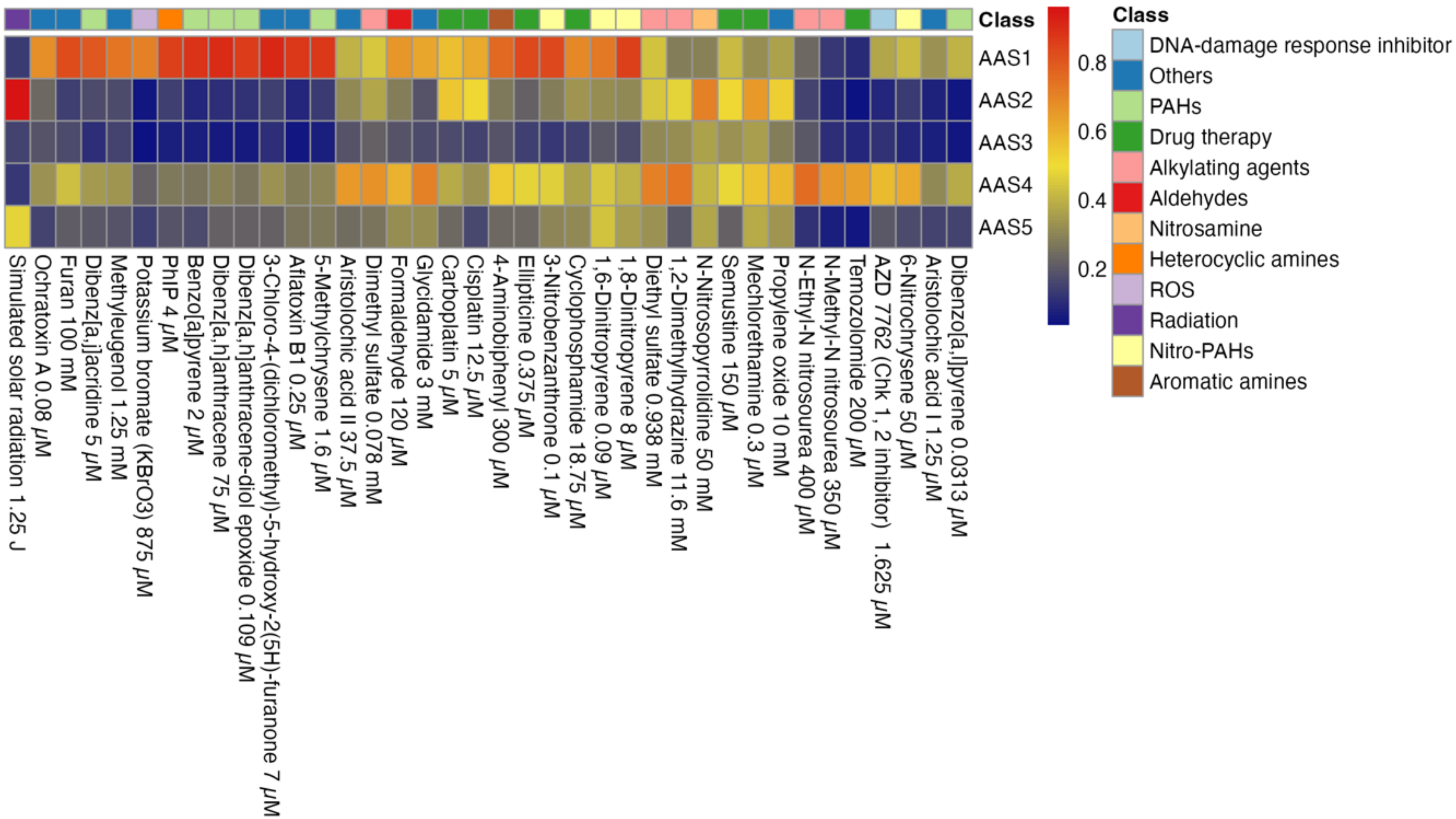

Appendix Figure S3.

The similarity between the distribution of amino acid substitutions associated with mutagens and AAS1-AAS5. The cosine similarity between AASs and the amino acid substitution frequency generated by different mutagens is shown color-coded. The columns (mutagens) are clustered using the Euclidean distance and the ward.D2 hierarchical clustering method. Each mutagen is classified to mutagen groups according to Kucab et al. Cell, 2019.

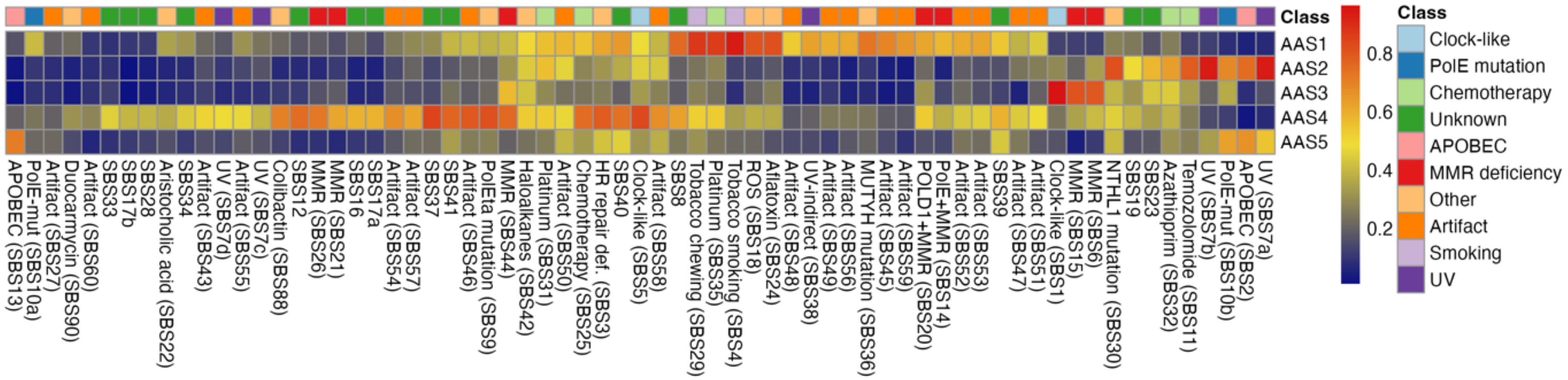

Appendix Figure S4.

The similarity between the distribution of amino acid substitutions associated with single base substitutions (SBS) and AAS1-AAS5. The cosine similarity between AASs and the amino acid substitution frequency associated with different SBS signatures is shown color-coded. The columns (SBSs) are clustered using the Euclidean distance and the ward.D2 hierarchical clustering method. Each mutagen is classified to mutagen groups according to the COSMIC website.

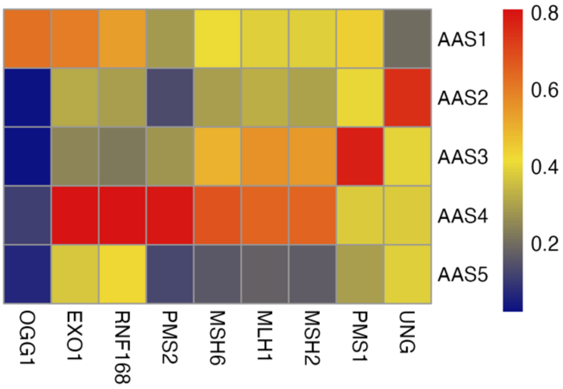

Appendix Figure S5.

The similarity between the distribution of amino acid substitutions associated with gene knockouts and AAS1-AAS5. The cosine similarity between AASs and the amino acid substitution frequency found in cell lines lacking certain DNA repair genes is shown color-coded. The columns (genes) are clustered using the Euclidean distance and the ward.D2 hierarchical clustering method.

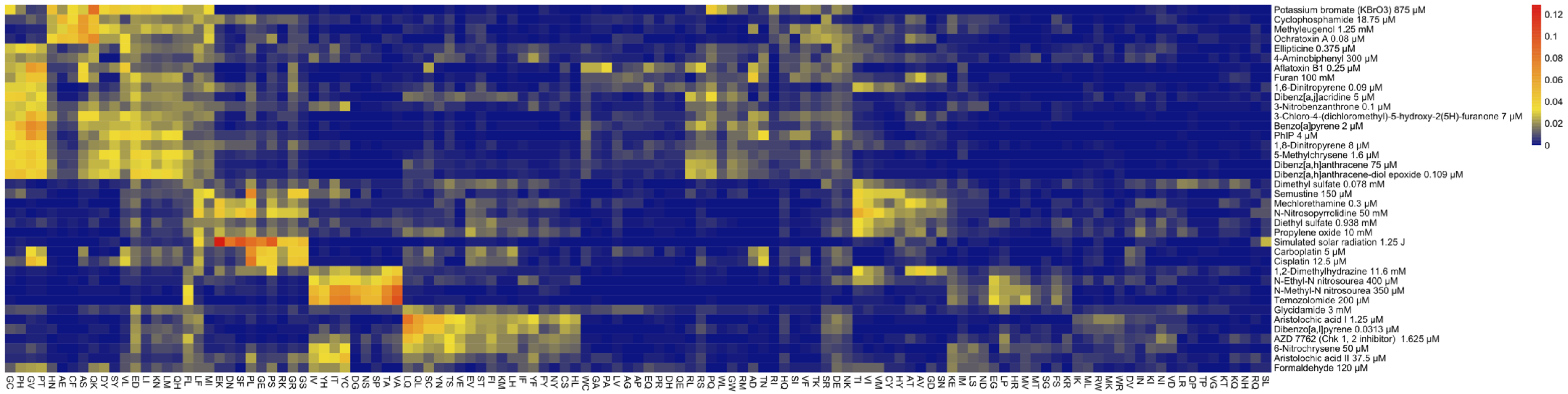

Appendix Figure S6.

The frequency of amino acid substitutions associated with various environmental mutagens. The heatmap indicates the relative frequency of amino acid substitutions generated in simulations color-coded. Substitutions reaching at least 1% frequency for any mutagens are indicated. The rows (mutagens) and columns (substitutions) are clustered using the Euclidean distance and the ward.D2 hierarchical clustering method. Each amino acid substitution is denoted by the single-letter codes of the original and mutated amino acids.

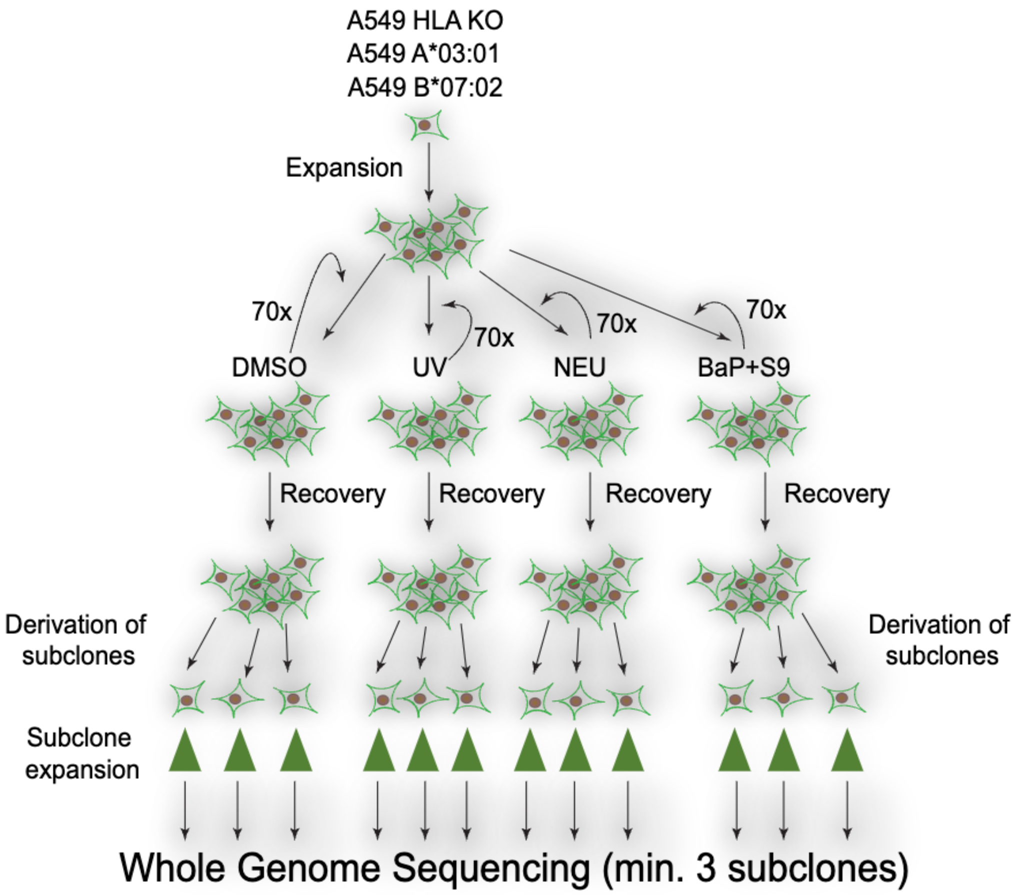

Appendix Figure S7.

Experimental workflow for inducing mutations. The schematic illustrates the experimental workflow for deriving and sequencing subclones from A549 cells expressing HLA-A*03:01 and HLA-B*07:02 or no HLA alleles (KO). After expansion, the cells were exposed to four different treatments: DMSO (solvent control), UV irradiation (10% UVB 295–315 nm and 90% UVA 315–400 nm, with a total dose of 1.25 J), N-ethyl-N-nitrosourea (NEU) at 400 uM, and benzo[a]pyrene at 0.39 uM with S9 metabolic activation (BaP+S9), each for 70 cycles. Following treatment and recovery, subclones were isolated and expanded. A minimum of three subclones from each condition were subjected to whole-genome sequencing to assess the mutational signatures induced by the different mutagenic treatments.

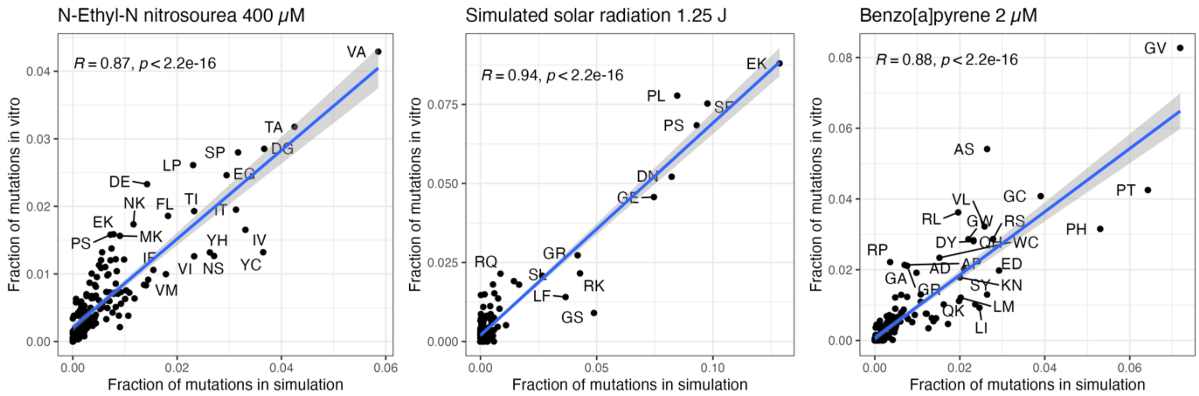

Appendix Figure S8.

Computational simulations reliably predict the distribution of amino acid substitutions induced by various mutagens. The relationship between the mean fraction of experimentally observed amino acid substitutions and values obtained through simulations is illustrated for three common mutagens (n = 166 substitutions). Pearson's correlation coefficients and p values of two-sided correlation tests are shown. Blue lines indicate linear regression line, while grey-shaded areas indicate 95% confidence interval. Each amino acid substitution is denoted by the single-letter codes of the original and mutated amino acids.

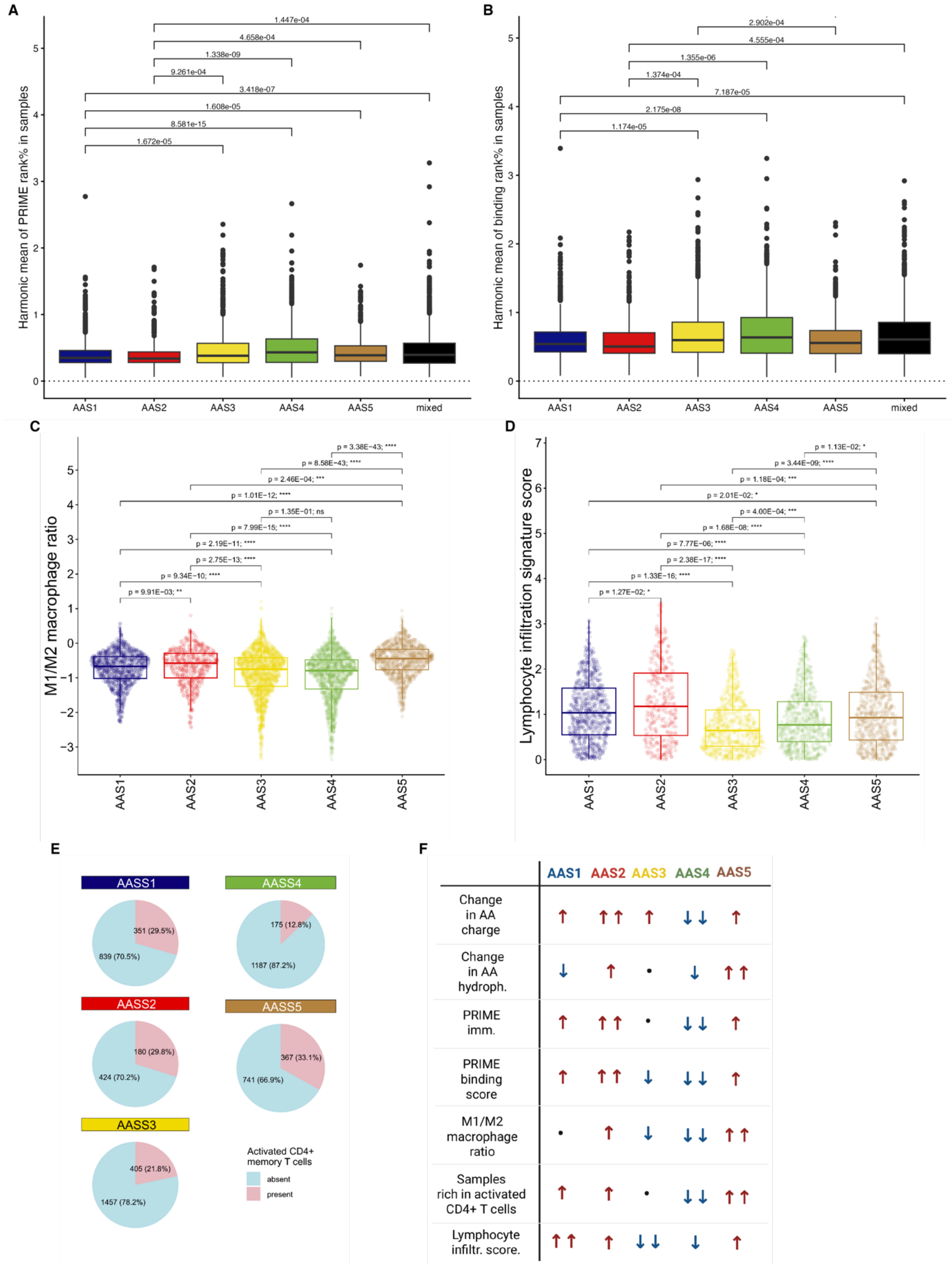

Appendix Figure S9.

AASs are associated with distinct effects on amino acid biophysical properties, neopeptide immunogenicity, and the tumour microenvironment A-B) AAS1 to AAS5 are associated with the formation of neopeptides with varying immunogenicity. The harmonic mean of PRIME rank% (A) and HLA binding rank% (B) values are shown in tumour samples dominated by different

signatures. Lower rank% values indicate higher immunogenicity (A) and stronger HLA binding (B). Kruskal–Wallis test p values are 4.6×10^{-17} and 9.8×10^{-14} for panels A and B, respectively; FDR-corrected p values from Dunn's tests are indicated above the horizontal segments for significant ($p < 0.05$) differences. C-D) Ratio of M1/M2 macrophages (C) and lymphocyte infiltration signature score (D) in samples belonging to different AAS groups. Kruskal–Wallis test $p < 2.2 \times 10^{-16}$. On boxplots, horizontal lines indicate median, boxes indicate the IQR, and whiskers indicate first quartile $-1.5 \times$ IQR and third quartile $+1.5 \times$ IQR. Outliers are not shown for visualization purposes. E) Number and fraction of samples with activated memory $CD4^{+}$ T cells among tumours dominated by AAS1–5. F) Summary of the findings shown in Figure 4A–B and in panels A–E of this appendix figure. AAS4 is characterized by exceptionally low changes in amino-acid charge and hydrophobicity, together with lower PRIME immunogenicity and binding scores, fewer $CD4^{+}$ T-cell–rich samples, and reduced lymphocyte infiltration. In contrast, AAS2 and AAS5 display strong increases in charge and hydrophobicity and are consistently associated with higher PRIME scores, a more pro-inflammatory M1/M2 macrophage ratio, more $CD4^{+}$ T-cell–rich samples, and higher lymphocyte infiltration. The patterns observed for AAS2 support the exceptional immunogenicity of UV-associated melanoma samples (see also Figure 2C, and 6B-C).

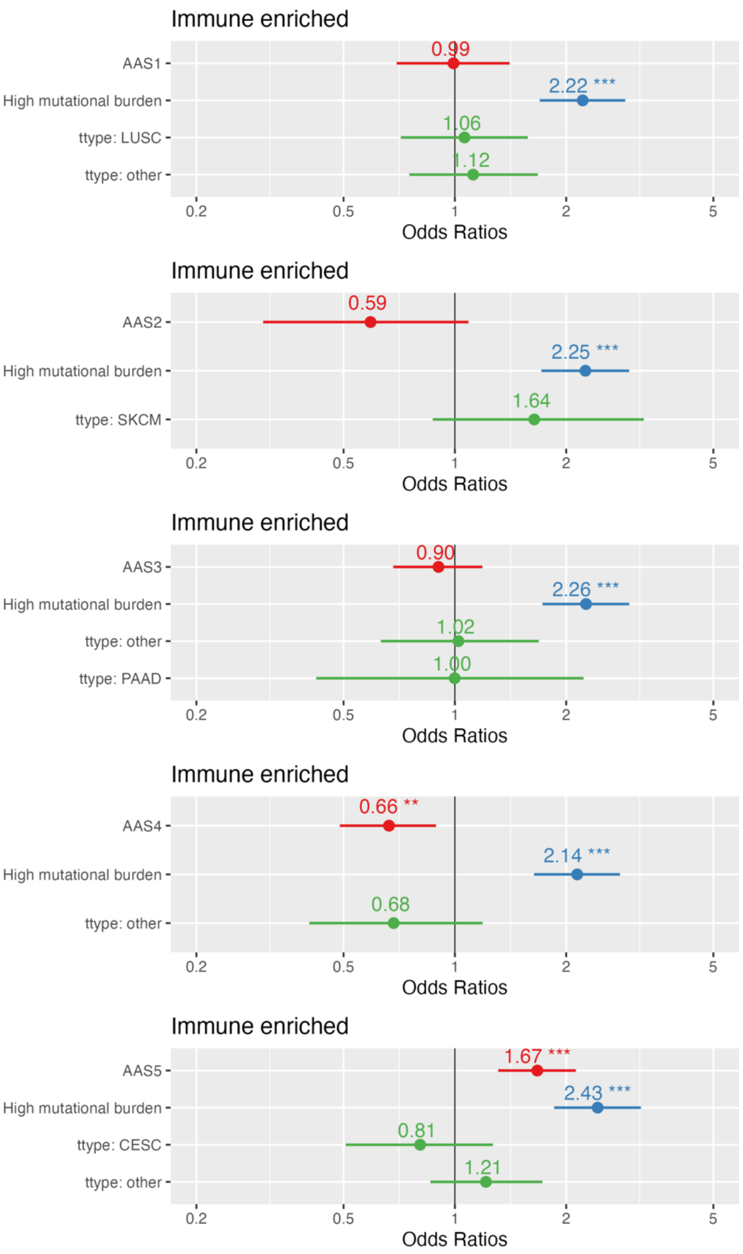

Appendix Figure S10.

The effect of AAS on immune phenotype is independent of TMB and tumor type. The forest plots display the coefficients and 95% confidence intervals for the predictor variables in the logistic regression models. Predictors with statistically significant effects are marked with asterisks. The models were constructed using the formula:

$$Immune\_enriched \sim AASn + High\ TMB + ttype\_1 + \ldots + ttype\_n,$$

where *Immune_enriched* is a categorical variable representing the immune-enriched phenotype category, *AASn* indicates the dominance of AAS1–5 in the samples, *ttype_1* to *ttype_n* represent various tumor types. Only tumor types with a median prevalence of the examined AAS greater than 0.7 were included as individual predictors. Tumor types not meeting this criterion were grouped into "other" category for the analysis. *High TMB* was defined as TMB belonging to the top 25% of all TMB values.

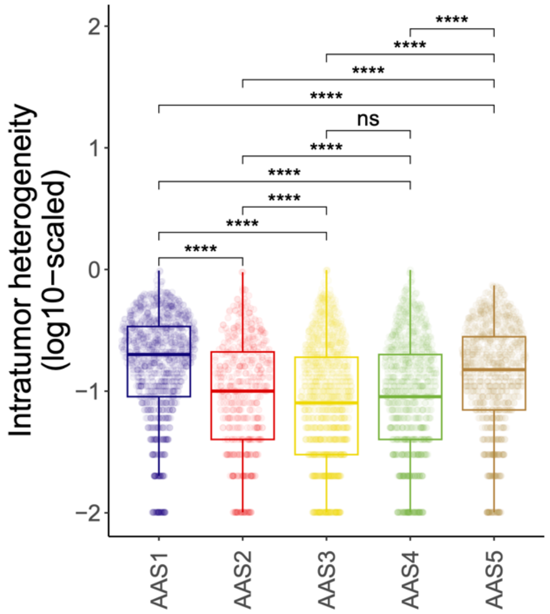

1st group	2nd group	p_{corr}
AAS1	AAS2	$6.08*10^{-25}$
AAS1	AAS3	$1.58*10^{-94}$
AAS2	AAS3	$3.85*10^{-7}$
AAS1	AAS4	$3.83*10^{-78}$
AAS2	AAS4	$4.66*10^{-6}$
AAS3	AAS4	0.32
AAS1	AAS5	4.75*10-6
AAS2	AAS5	$1.11*10^{-10}$
AAS3	AAS5	$9.1*10^{-50}$
AAS4	AAS5	3.39*10-41

Appendix Figure S11.

AAS4-dominated tumors have lower intratumor heterogeneity (ITH) than samples dominated by AAS1, 2 and 5. Kruskal-Wallis p value is lower than $2.2*10^{-16}$. On the boxplot, horizontal lines indicate median, boxes indicate the IQR, and whiskers indicate first quartile – 1.5 × IQR and third quartile +1.5 × IQR. Samples with ITH = 0 are not shown because the y-axis is log-transformed, but they were included in statistical analyses. NS: non-significant. ****: $p<10^{-4}$. The FDR-corrected p values of a Dunn's post-hoc test are shown in the table.

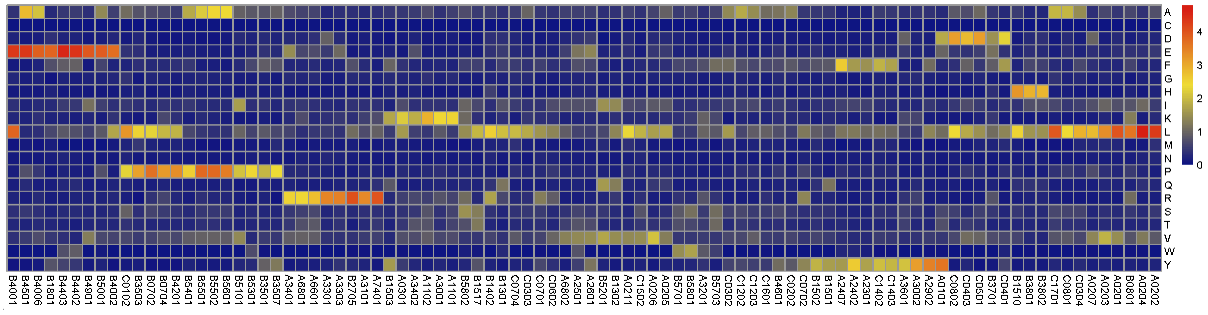

Appendix Figure S12.

The affinity of HLA alleles for the 20 amino acids. The values representing amino acid preferences HLA alleles are shown color-coded. The columns (alleles) are clustered using the Euclidean distance and the ward.D2 hierarchical clustering method.

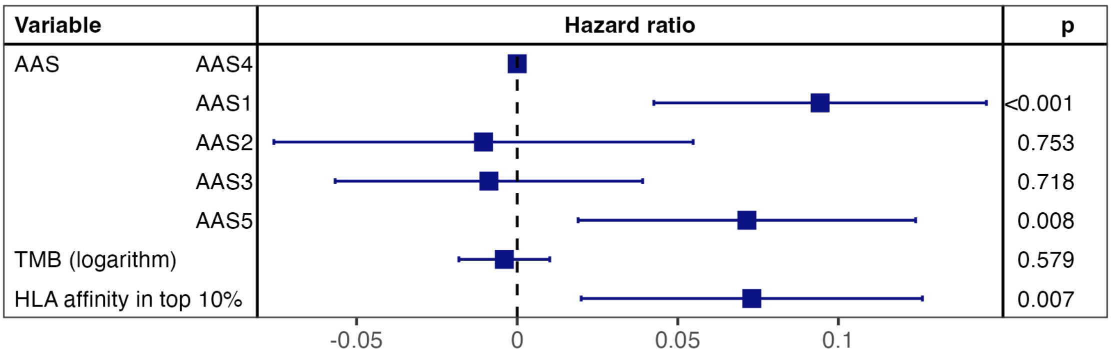

Appendix Figure S13.

AASs and HLA binding determines immune selection in cancer. The summary of a multivariate logistic regression model is indicated. The model was constructed using the formula:

$$Immune\_selected \sim AASn + log(TMB) + high\_HLA\_aff,$$

where *Immune_selected* is a binary variable indicating immune dN/dS < 0.82 (n = 1273 immune-selected and 2208 non-immune-selected samples), *AASn* denotes the dominant AAS category in each sample (AAS1-AAS5; n = 788, 353, 993, 652 and 695, respectively). log(TMB) is the natural logarithm of the tumour mutational burden, and *high_HLA-aff* is a binary indicator for samples in the top 10% of the cohort when ranked by the predicted binding affinity of their HLA molecules to the AASs present in the same sample (n = 454 in-group and 4,154 out-group samples) . Blue squares indicate coefficients associated with each independent variable, while blue horizontal lines indicate 95% confidence interval.

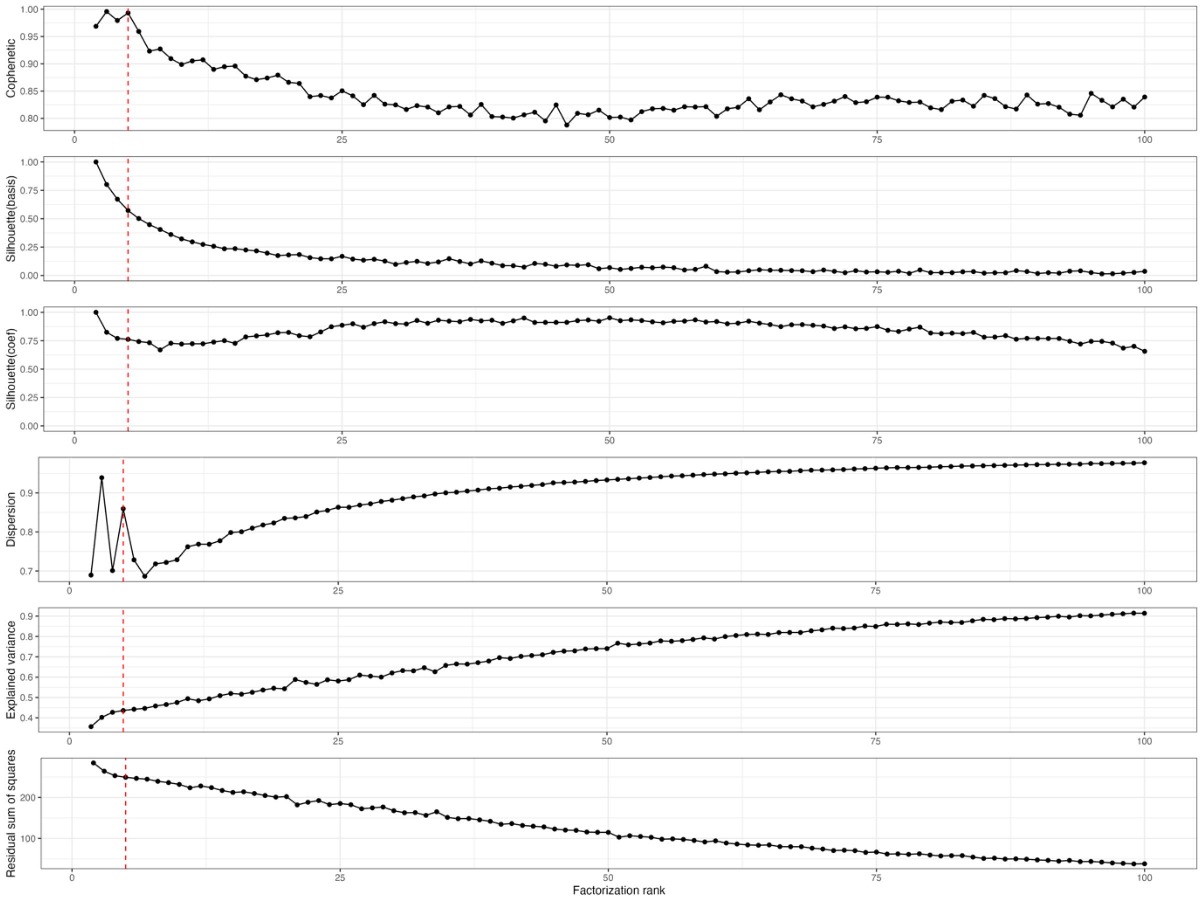

Appendix Figure S14.

The cophenetic, silhouette width, dispersion, explained variance and residual sum of square measures are indicated when carrying out NMF using different ranks. The red dashed line indicates the chosen factor of 5.

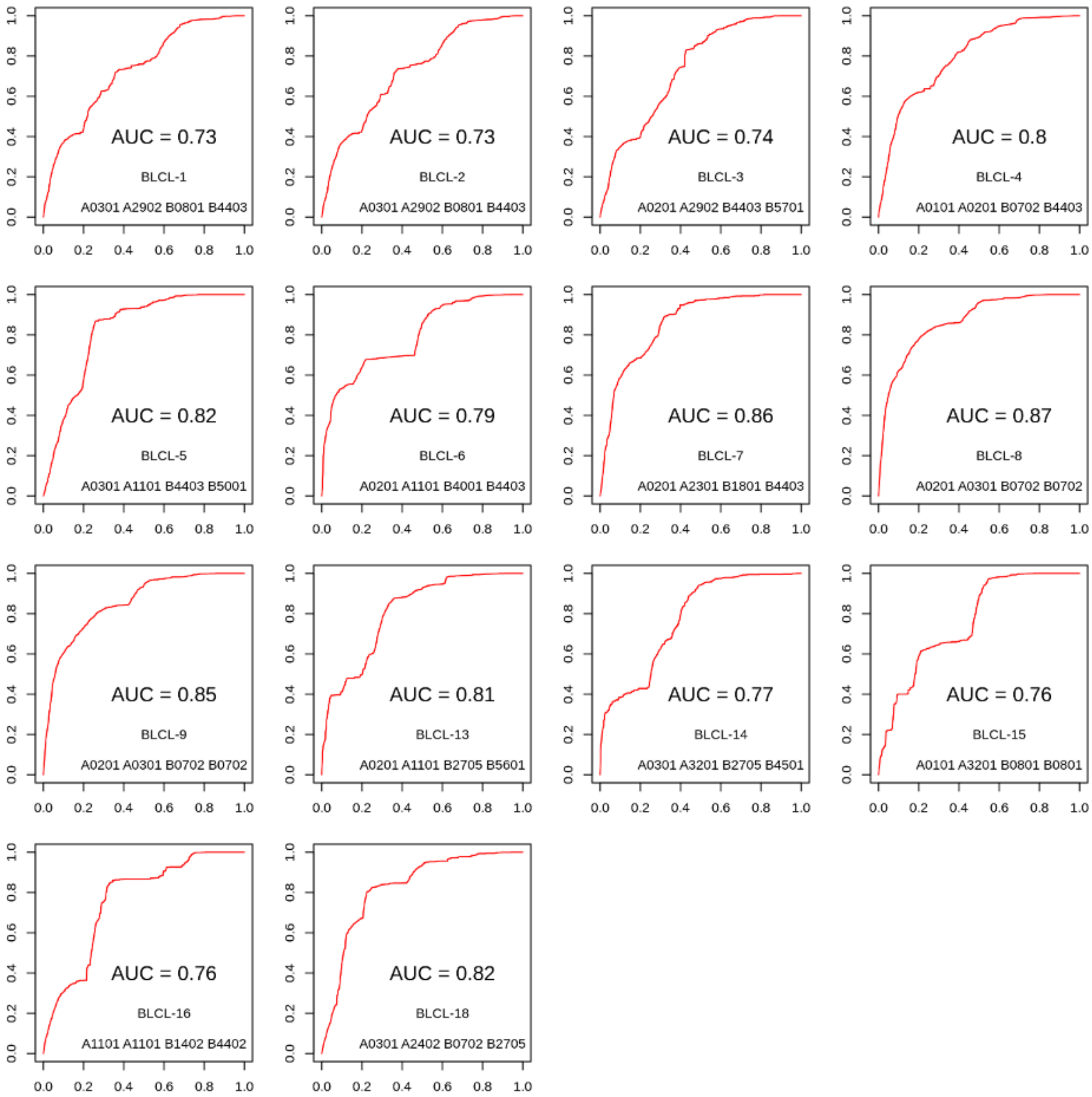

Appendix Figure S15.

The calculated amino acid specificity values are accurate. Area under the ROC curve is shown for different samples. The HLA-A and B alleles of each sample are also indicated. See Methods for detailed explanation.

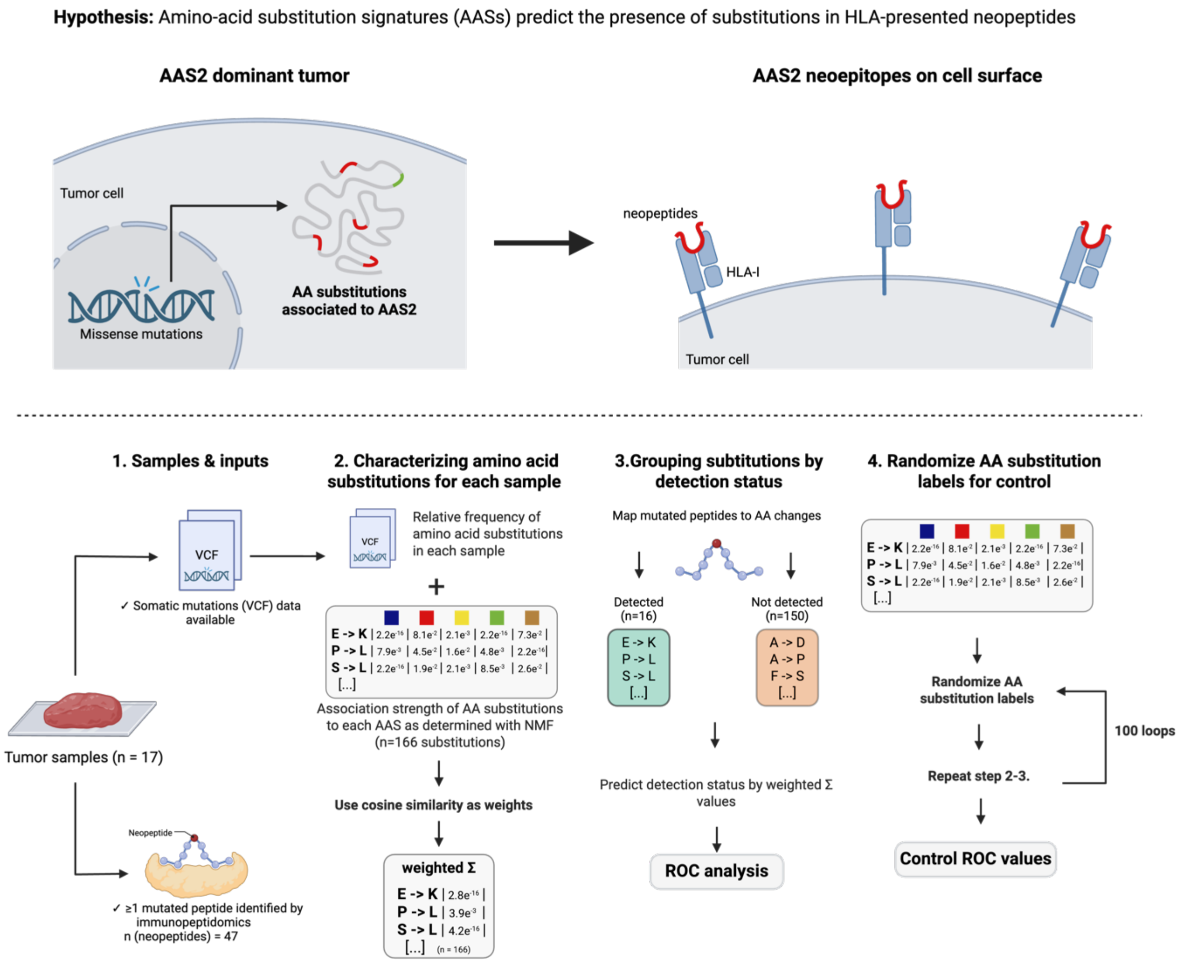

Appendix Figure S16.

Conceptual overview illustrating the rationale and workflow of the analysis performed to validate AAS signatures using immunopeptidomics data. See the main text, Methods, and Appendix Supplementary Text for details.

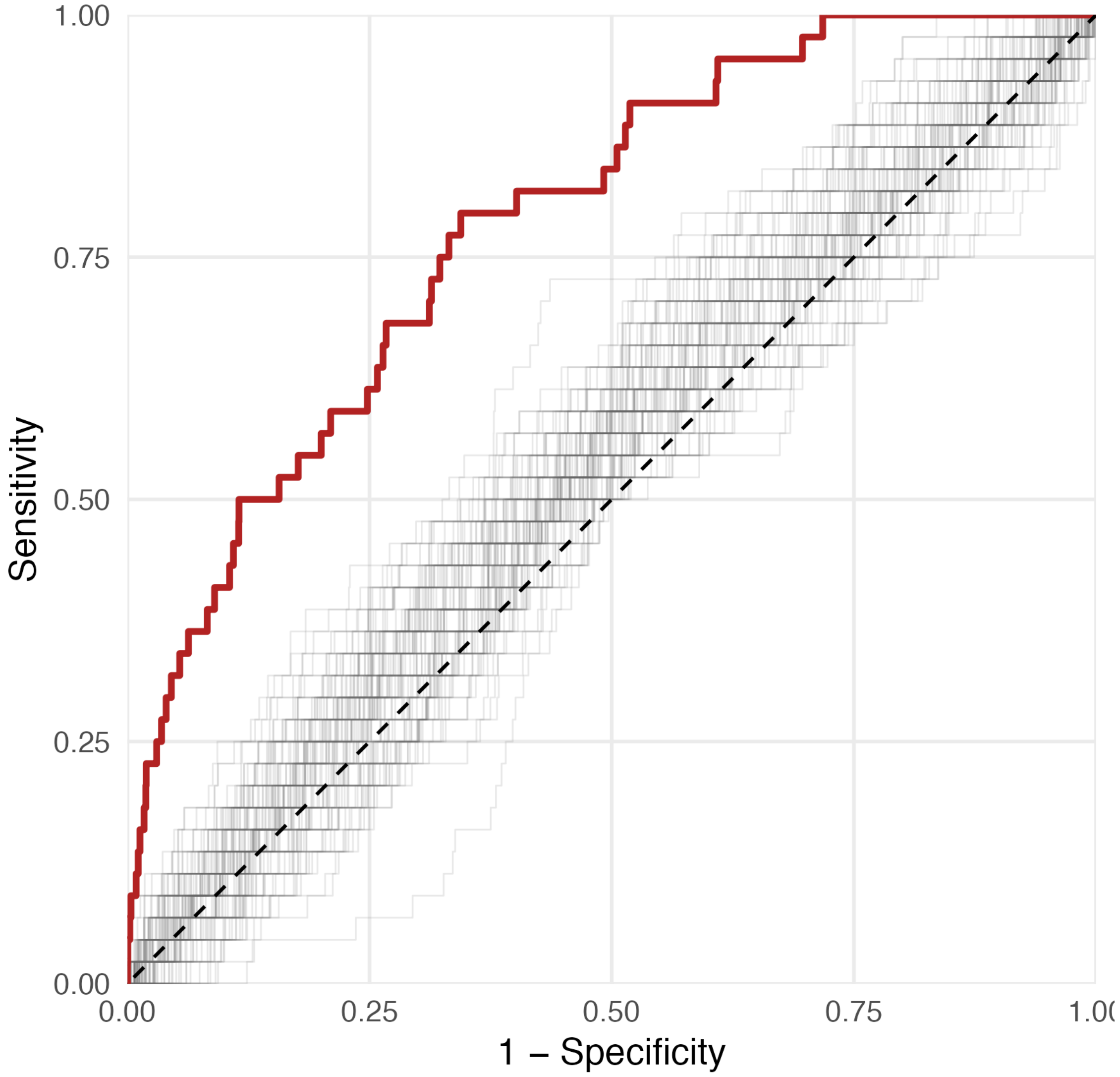

Appendix Figure S17.

Influence of AASs on the immunopeptidome. The ROC curve showing how well the calculated association score values in tumor samples predict the presence of amino acid substitutions in immunopeptidomics data (AUC = 0.785). For comparison, 100 ROC curves obtained after randomizing amino acid substitution labels in the AAS coefficient matrix are shown in grey (with increased transparency for clarity, AUC min: 0.445, max: 0.612, mean: 0.535, SD: 0.033).

ID	Treatment	HLA	down-stream	exonic	exonic splicing	intergenic	intronic	ncRNA exonic	ncRNA exonic splicing	ncRNA intronic	ncRNA splicing	splicing	upstream	upstream down-stream	UTR3	UTR5	UTR5 UTR3	frameshift deletion	frameshift insertion	non-frameshift deletion	nonsyn SNV	stop-gain	stop-loss	syn. SNV	unknown	total	deletions	insertions
A2NT3	non-treated	A*03*01	17 (0.49%)	46 (1.34%)	0 (0%)	1942 (56.4%)	1154 (33.52%)	9 (0.26%)	0 (0%)	175 (5.08%)	0 (0%)	3 (0.09%)	38 (1.1%)	1 (0.03%)	35 (1.02%)	23 (0.67%)	0 (0%)	0 (0%)	0 (0%)	0 (0%)	38 (82.61%)	1 (2.17%)	0 (0%)	7 (15.22%)	0 (0%)	3422	15	6
B4NT3	non-treated	B*07:02	14 (0.41%)	53 (1.53%)	0 (0%)	1942 (56.19%)	1115 (32.26%)	11 (0.32%)	0 (0%)	236 (6.83%)	0 (0%)	2 (0.06%)	26 (0.75%)	1 (0.03%)	39 (1.13%)	17 (0.49%)	0 (0%)	0 (0%)	0 (0%)	0 (0%)	38 (71.7%)	2 (3.77%)	0 (0%)	13 (24.53%)	0 (0%)	3427	19	10
KONT3	non-treated	knock-out	23 (0.61%)	54 (1.43%)	0 (0%)	2139 (56.54%)	1248 (32.99%)	19 (0.5%)	0 (0%)	215 (5.68%)	0 (0%)	2 (0.05%)	29 (0.77%)	1 (0.03%)	35 (0.93%)	18 (0.48%)	0 (0%)	1 (1.85%)	0 (0%)	0 (0%)	41 (75.93%)	4 (7.41%)	0 (0%)	7 (12.96%)	1 (1.85%)	3751	22	10
BAP1A3	Benzopyren	A*03*01	431 (0.63%)	981 (1.43%)	0 (0%)	38681 (56.55%)	22106 (32.32%)	310 (0.45%)	0 (0%)	4426 (6.47%)	5 (0.01%)	23 (0.03%)	664 (0.97%)	25 (0.04%)	499 (0.73%)	256 (0.37%)	0 (0%)	20 (2.04%)	2 (0.2%)	0 (0%)	676 (68.91%)	54 (5.5%)	0 (0%)	226 (23.04%)	3 (0.31%)	66229	1921	257
BAP2A7	Benzopyren	A*03*01	592 (0.58%)	1361 (1.34%)	0 (0%)	57823 (56.95%)	32523 (32.03%)	447 (0.44%)	1 (0%)	6453 (6.36%)	5 (0%)	31 (0.03%)	969 (0.95%)	43 (0.04%)	895 (0.88%)	380 (0.37%)	3 (0%)	33 (2.42%)	7 (0.51%)	0 (0%)	928 (68.19%)	86 (6.32%)	1 (0.07%)	300 (22.04%)	6 (0.44%)	98133	2915	478
BAP1A8	Benzopyren	A*03*01	430 (0.63%)	938 (1.38%)	0 (0%)	38444 (56.6%)	21892 (32.23%)	316 (0.47%)	0 (0%)	4275 (6.29%)	0 (0%)	28 (0.04%)	727 (1.07%)	16 (0.02%)	588 (0.87%)	265 (0.39%)	4 (0.01%)	22 (2.35%)	2 (0.21%)	0 (0%)	641 (68.34%)	58 (6.18%)	0 (0%)	210 (22.39%)	5 (0.53%)	65733	1927	263
BAP1B1	Benzopyren	B*07:02	543 (0.67%)	1130 (1.4%)	0 (0%)	45647 (56.36%)	26167 (32.31%)	341 (0.42%)	0 (0%)	5224 (6.45%)	3 (0%)	28 (0.03%)	877 (1.08%)	34 (0.04%)	688 (0.85%)	315 (0.39%)	1 (0%)	24 (2.12%)	3 (0.27%)	0 (0%)	813 (71.95%)	45 (3.98%)	2 (0.18%)	237 (20.97%)	6 (0.53%)	78512	2180	306
BAP1B3	Benzopyren	B*07:02	452 (0.68%)	948 (1.42%)	0 (0%)	37341 (55.84%)	21944 (32.81%)	297 (0.44%)	0 (0%)	4303 (6.43%)	4 (0.01%)	24 (0.04%)	686 (1.03%)	18 (0.03%)	590 (0.88%)	265 (0.4%)	1 (0%)	17 (1.79%)	1 (0.11%)	0 (0%)	674 (71.1%)	43 (4.54%)	1 (0.11%)	207 (21.84%)	5 (0.53%)	64580	2003	290
BAP2B1	Benzopyren	A*03*01	397 (0.62%)	815 (1.28%)	0 (0%)	36237 (56.84%)	20529 (32.2%)	301 (0.47%)	0 (0%)	4072 (6.39%)	2 (0%)	22 (0.03%)	612 (0.96%)	19 (0.03%)	518 (0.81%)	233 (0.37%)	1 (0%)	21 (2.58%)	0 (0%)	0 (0%)	550 (67.48%)	45 (5.52%)	1 (0.12%)	193 (23.68%)	5 (0.61%)	61526	1979	253
BAP1KO3	Benzopyren	knock-out	387 (0.62%)	814 (1.31%)	2 (0%)	35460 (56.98%)	20065 (32.24%)	262 (0.42%)	0 (0%)	3876 (6.23%)	2 (0%)	29 (0.05%)	564 (0.91%)	19 (0.03%)	503 (0.81%)	253 (0.41%)	0 (0%)	16 (1.96%)	2 (0.25%)	0 (0%)	564 (69.12%)	37 (4.53%)	0 (0%)	196 (24.02%)	1 (0.12%)	60186	1774	276
BAP2KO2	Benzopyren	knock-out	538 (0.66%)	1069 (1.32%)	3 (0%)	46134 (56.92%)	25806 (31.84%)	386 (0.48%)	0 (0%)	5211 (6.43%)	2 (0%)	31 (0.04%)	783 (0.97%)	32 (0.04%)	744 (0.92%)	308 (0.38%)	0 (0%)	25 (2.33%)	2 (0.19%)	0 (0%)	735 (68.56%)	59 (5.5%)	0 (0%)	249 (23.23%)	2 (0.19%)	78569	2141	337
NEU1A6	N-nitroso-N-methylurea	A*03:01	2907 (0.6%)	4898 (1.01%)	4 (0%)	263247 (54.29%)	172023 (35.48%)	1786 (0.37%)	0 (0%)	31392 (6.47%)	17 (0%)	125 (0.03%)	2838 (0.59%)	98 (0.02%)	4823 (0.99%)	733 (0.15%)	4 (0%)	0 (0%)	0 (0%)	0 (0%)	3455 (70.48%)	203 (4.14%)	13 (0.27%)	1213 (24.75%)	18 (0.37%)	484723	132	40
NEU2A5	N-nitroso-N-methylurea	A*03:01	2263 (0.58%)	3912 (1.01%)	3 (0%)	211306 (54.4%)	136656 (35.18%)	1508 (0.39%)	0 (0%)	25807 (6.64%)	14 (0%)	121 (0.03%)	2331 (0.6%)	110 (0.03%)	3799 (0.98%)	613 (0.16%)	3 (0%)	0 (0%)	0 (0%)	0 (0%)	2715 (69.35%)	178 (4.55%)	5 (0.13%)	998 (25.49%)	19 (0.49%)	388287	132	27
NEU2A6	N-nitroso-N-methylurea	A*03:01	2866 (0.61%)	4821 (1.03%)	2 (0%)	252887 (53.83%)	167986 (35.76%)	1748 (0.37%)	1 (0%)	30982 (6.59%)	17 (0%)	150 (0.03%)	2742 (0.58%)	105 (0.02%)	4727 (1.01%)	779 (0.17%)	3 (0%)	0 (0%)	0 (0%)	1 (0.02%)	3392 (70.33%)	183 (3.79%)	10 (0.21%)	1218 (25.25%)	19 (0.39%)	469655	118	43
NEU1B4	N-nitroso-N-methylurea	B*07:02	1147 (0.56%)	2024 (0.99%)	3 (0%)	112429 (54.99%)	71492 (34.97%)	752 (0.37%)	0 (0%)	13077 (6.4%)	4 (0%)	56 (0.03%)	1135 (0.56%)	33 (0.02%)	1982 (0.97%)	299 (0.15%)	2 (0%)	1 (0.05%)	0 (0%)	0 (0%)	1414 (69.76%)	76 (3.75%)	5 (0.25%)	526 (25.95%)	5 (0.25%)	204349	67	19
NEU1B6	N-nitroso-N-methylurea	B*07:02	741 (0.56%)	1227 (0.92%)	2 (0%)	73658 (55.47%)	46065 (34.69%)	441 (0.33%)	1 (0%)	8400 (6.33%)	3 (0%)	40 (0.03%)	710 (0.53%)	24 (0.02%)	1265 (0.95%)	207 (0.16%)	0 (0%)	1 (0.08%)	0 (0%)	0 (0%)	859 (69.89%)	45 (3.66%)	4 (0.33%)	317 (25.79%)	3 (0.24%)	132742	38	4
NEU2B5	N-nitroso-N-methylurea	B*07:02	1203 (0.57%)	1984 (0.95%)	0 (0%)	114582 (54.75%)	73463 (35.1%)	751 (0.36%)	1 (0%)	13626 (6.51%)	12 (0.01%)	62 (0.03%)	1230 (0.59%)	36 (0.02%)	2006 (0.96%)	312 (0.15%)	2 (0%)	1 (0.05%)	0 (0%)	0 (0%)	1400 (70.56%)	79 (3.98%)	2 (0.1%)	498 (25.1%)	4 (0.2%)	209208	45	17
NEU1KO1	N-nitroso-N-methylurea	knock-out	1602 (0.59%)	2842 (1.05%)	0 (0%)	146664 (54.33%)	95329 (35.31%)	1044 (0.39%)	1 (0%)	17872 (6.62%)	10 (0%)	81 (0.03%)	1501 (0.56%)	51 (0.02%)	2577 (0.95%)	394 (0.15%)	3 (0%)	0 (0%)	0 (0%)	1 (0.04%)	1958 (68.9%)	111 (3.91%)	7 (0.25%)	753 (26.5%)	12 (0.42%)	269872	81	18
NEU1KO2	N-nitroso-N-methylurea	knock-out	1431 (0.59%)	2323 (0.96%)	2 (0%)	134055 (55.15%)	84708 (34.85%)	911 (0.37%)	0 (0%)	15596 (6.42%)	9 (0%)	69 (0.03%)	1329 (0.55%)	46 (0.02%)	2237 (0.92%)	366 (0.15%)	3 (0%)	1 (0.04%)	0 (0%)	0 (0%)	1653 (71.1%)	90 (3.87%)	5 (0.22%)	570 (24.52%)	6 (0.26%)	242994	67	24
NEU2KO5	N-nitroso-N-methylurea	knock-out	1917 (0.58%)	3227 (0.98%)	1 (0%)	181042 (54.76%)	115961 (35.07%)	1246 (0.38%)	0 (0%)	21690 (6.56%)	7 (0%)	81 (0.02%)	1838 (0.56%)	56 (0.02%)	3090 (0.93%)	464 (0.14%)	2 (0%)	0 (0%)	0 (0%)	0 (0%)	2251 (69.73%)	120 (3.72%)	9 (0.28%)	841 (26.05%)	7 (0.22%)	330494	104	24
UV1KO2	UV	knock-out	95 (0.45%)	182 (0.87%)	0 (0%)	12675 (60.36%)	6325 (30.12%)	57 (0.27%)	0 (0%)	1322 (6.3%)	0 (0%)	4 (0.02%)	137 (0.65%)	3 (0.01%)	169 (0.8%)	31 (0.15%)	0 (0%)	0 (0%)	0 (0%)	0 (0%)	125 (68.68%)	6 (3.3%)	0 (0%)	50 (27.47%)	1 (0.55%)	20859	122	19
UV1KO3	UV	knock-out	117 (0.51%)	173 (0.76%)	0 (0%)	13201 (57.99%)	7294 (32.04%)	89 (0.39%)	0 (0%)	1490 (6.55%)	1 (0%)	3 (0.01%)	149 (0.65%)	6 (0.03%)	192 (0.84%)	48 (0.21%)	0 (0%)	1 (0.58%)	0 (0%)	0 (0%)	100 (57.8%)	5 (2.89%)	2 (1.16%)	64 (36.99%)	1 (0.58%)	22550	194	19
UV3UV1	UV	A*03:01	83 (0.49%)	145 (0.85%)	0 (0%)	10236 (60%)	5220 (30.6%)	39 (0.23%)	0 (0%)	1108 (6.49%)	0 (0%)	0 (0%)	84 (0.49%)	4 (0.02%)	117 (0.69%)	24 (0.14%)	0 (0%)	1 (0.69%)	0 (0%)	0 (0%)	92 (63.45%)	12 (8.28%)	0 (0%)	40 (27.59%)	0 (0%)	16935	106	19
UV4UV1	UV	A*03:01	149 (0.5%)	220 (0.74%)	0 (0%)	18037 (60.4%)	8929 (29.9%)	91 (0.3%)	0 (0%)	1960 (6.56%)	0 (0%)	5 (0.02%)	179 (0.6%)	8 (0.03%)	224 (0.75%)	60 (0.2%)	0 (0%)	1 (0.45%)	0 (0%)	0 (0%)	143 (65%)	8 (3.64%)	0 (0%)	68 (30.91%)	0 (0%)	29631	201	30
UV4UV2	UV	A*03:01	80 (0.45%)	126 (0.71%)	0 (0%)	10792 (60.73%)	5277 (29.7%)	67 (0.38%)	0 (0%)	1194 (6.72%)	1 (0.01%)	4 (0.02%)	79 (0.44%)	3 (0.02%)	117 (0.66%)	29 (0.16%)	0 (0%)	0 (0%)	0 (0%)	0 (0%)	75 (59.52%)	7 (5.56%)	0 (0%)	43 (34.13%)	1 (0.79%)	17634	119	16

Appendix Table S1.

The number and fraction of mutations generated in A549 cells. The classification of all mutations (green) and exonic mutations (red) are also highlighted. The fraction of mutations is calculated separately for the two classifications. Samples used in T cell proliferation assays are highlighted with red color.

Comparison	Corrected p value for hydrophobicity change	Corrected p value for change in charge
AAS1 - AAS2	$7.34*10^{-18}$	$5.68*10^{-186}$
AAS1 - AAS3	$2.1*10^{-26}$	$2.13*10^{-127}$
AAS2 - AAS3	0.17	$6.17*10^{-36}$
AAS1 - AAS4	$7.15*10^{-73}$	0.001
AAS2 - AAS4	$3.89*10^{-116}$	$1.58*10^{-161}$
AAS3 - AAS4	$5.02*10^{-209}$	$1.65*10^{-100}$
AAS1 - AAS5	$2.85*10^{-08}$	0
AAS2 - AAS5	$6.04*10^{-5}$	$2.71*10^{-13}$
AAS3 - AAS5	$3.2*10^{-5}$	$4.08*10^{-139}$
AAS4 - AAS5	$1.25*10^{-114}$	0
AAS1 - mixed	0.002	$4.24*10^{-66}$
AAS2 - mixed	$1.3*10^{-30}$	$2.5*10^{-76}$
AAS3 - mixed	$1.71*10^{-56}$	$2.21*10^{-18}$
AAS4 - mixed	$1.53*10^{-69}$	$1.12*10^{-45}$
AAS5 - mixed	$3.18*10^{-19}$	$1.09*10^{-238}$

Appendix Table S2.

Corrected p values of Dunn's tests carried out for Figure 4A and B. The p values were corrected using the Benjamini-Hochberg method.

Immune marker	Comparison	Difference	Lower CI	Upper CI	Adjusted p value
**M1/M2 macrophage ratio**	AAS4-others	-0.22	-0.38	-0.056	0.005
	AAS5-others	0.31	0.14	0.49	$9.92*10^{-5}$
	AAS5-AAS4	0.53	0.32	0.74	$6.67*10^{-9}$
**Lymphocyte infiltration signature score**	AAS4-others	-0.15	-0.24	-0.05	$6.12*10^{-4}$
	AAS5-others	0.29	0.19	0.39	$7.08*10^{-12}$
	AAS5-AAS4	0.44	0.32	0.56	≈0
**Activated memory CD4+ T cells**	AAS4-others	-0.55	-0.68	-0.42	$9.84*10^{-13}$
	AAS5-others	0.31	0.17	0.45	$3.95*10^{-7}$
	AAS5-AAS4	0.86	0.7	1.03	$9.56*10^{-13}$

Appendix Table S3.

Pairwise comparison of AAS groups using various immune markers after controlling for covariates. The table presents the results of Tukey's Honest Significant Difference (HSD) post hoc test, following an analysis of variance (ANOVA) on several immune markers (obtained from Thorsson et al., Immunity, 2018) to assess differences across groups based on dominant amino acid substitution signatures (AAS4, AAS5, and other AASs). The ANOVA model includes a binary indicator of high TMB and categorical tumor types as covariates, enabling the evaluation of AAS groups while controlling for these factors. High TMB was defined as TMB belonging to the top 25% of all TMB values. Lower and upper CI are the lower and upper bounds of the 95% confidence interval for the estimated mean difference.

Comparison	p value	Corr. p value
BEAD, PBMC: B - CTRL, PBMC: B	$3.48*10^{-6}$	$1.6*10^{-4}$
BEAD, PBMC: A - CTRL, PBMC: B	$1.58*10^{-5}$	$3.6*10^{-4}$
BEAD, PBMC: B - Cell: KO, PBMC: B	$9.34*10^{-5}$	0.001
BEAD, PBMC: A - Cell: KO, PBMC: B	$1.99*10^{-4}$	0.001
BEAD, PBMC: A - CTRL, PBMC: A	$1\text{-}98*10^{-4}$	0.002
BEAD, PBMC: B - CTRL, PBMC: A	$1.59*10^{-4}$	0.002
Cell: B, PBMC: B - CTRL, PBMC: B	$4.26*10^{-4}$	0.003
BEAD, PBMC: A - Cell: A, PBMC: B	0.003	0.014
BEAD, PBMC: B - Cell: A, PBMC: B	0.003	0.014
BEAD, PBMC: A - Cell: KO, PBMC: A	0.004	0.017
BEAD, PBMC: B - Cell: KO, PBMC: A	0.005	0.017
BEAD, PBMC: A - Cell: B, PBMC: A	0.005	0.018
Cell: B, PBMC: B - CTRL, PBMC: A	0.004	0.018
Cell: B, PBMC: B - Cell: KO, PBMC: B	0.005	0.019
BEAD, PBMC: B - Cell: B, PBMC: A	0.007	0.02
Cell: A, PBMC: A - CTRL, PBMC: B	0.012	0.033
Cell: A, PBMC: A - CTRL, PBMC: A	0.029	0.075
BEAD, PBMC: A - Cell: A, PBMC: A	0.051	0.108
Cell: B, PBMC: B - Cell: KO, PBMC: A	0.055	0.112
Cell: A, PBMC: B - Cell: B, PBMC: B	0.05	0.112
Cell: A, PBMC: B - CTRL, PBMC: B	0.046	0.114
Cell: A, PBMC: A - Cell: KO, PBMC: B	0.05	0.118
Cell: B, PBMC: A - Cell: B, PBMC: B	0.062	0.123
BEAD, PBMC: A - Cell: B, PBMC: B	0.075	0.141
BEAD, PBMC: B - Cell: A, PBMC: A	0.08	0.145
Cell: A, PBMC: B - CTRL, PBMC: A	0.095	0.165
Cell: B, PBMC: A - CTRL, PBMC: B	0.117	0.194
BEAD, PBMC: B - Cell: B, PBMC: B	0.123	0.198
Cell: KO, PBMC: A - CTRL, PBMC: B	0.131	0.203
Cell: KO, PBMC: A - CTRL, PBMC: A	0.181	0.24
Cell: A, PBMC: B - Cell: KO, PBMC: B	0.176	0.24
Cell: B, PBMC: A - CTRL, PBMC: A	0.166	0.240
Cell: A, PBMC: A - Cell: A, PBMC: B	0.187	0.241
Cell: A, PBMC: A - Cell: KO, PBMC: A	0.161	0.241
Cell: A, PBMC: A - Cell: B, PBMC: A	0.176	0.247
Cell: KO, PBMC: B - CTRL, PBMC: B	0.224	0.28
Cell: B, PBMC: A - Cell: KO, PBMC: B	0.283	0.345
Cell: KO, PBMC: B - CTRL, PBMC: A	0.291	0.345
BEAD, PBMC: A - BEAD, PBMC: B	0.312	0.351
Cell: KO, PBMC: A - Cell: KO, PBMC: B	0.308	0.355
Cell: A, PBMC: A - Cell: B, PBMC: B	0.324	0.356
Cell: A, PBMC: B - Cell: KO, PBMC: A	0.399	0.427
Cell: A, PBMC: B - Cell: B, PBMC: A	0.426	0.446
Cell: B, PBMC: A - Cell: KO, PBMC: A	0.476	0.476
CTRL, PBMC: A - CTRL, PBMC: B	0.472	0.483

Appendix Table S4.

Results of Dunn's post-hoc test for Figure 7D. Raw and FDR-corrected p values are indicated. Most relevant groups with corrected p values < 0.2 are highlighted with different text colors: red: positive control; blue: negative control; green: both A549 cells and PBMCs carry the HLA-B*07:02 allele.

AAS	>25	>50
**AAS1**	0.99	0.979
**AAS2**	0.998	0.996
**AAS3**	0.997	0.988
**AAS4**	0.977	0.95
**AAS5**	0.997	0.993

Appendix Table S5.

AASs are independent of the selected mutation count cutoff. The table shows the cosine similarity of substitution prevalence for AAS1–5 when applying mutation count cutoffs of 25 and 50, compared with a cutoff of 10.

Sample	Total	Duplicate	Mapped	Properly mapped	PE mapped	SE mapped	With mate mapped to a different chr	With mate mapped to a different chr ((mapQ>=5))	Average sequencing depth	Coverage	Coverage at least 4X	Coverage at least 10X	Coverage at least 20X
**UV4UV1**	599381850 (100%)	129701553 (21.67%)	598545402 (99.86%)	588678228 (98.21%)	598088112 (99.78%)	914580 (0.15%)	5556536 (0.93%)	2831953 (0.47%)	29.99	99.69%	99.35%	98.01%	80.75%
**NEU1A6**	714089282 (100%)	194557421 (27.28%)	713083750 (99.86%)	702198728 (98.33%)	712584194 (99.79%)	999112 (0.14%)	5953830 (0.83%)	2771282 (0.39%)	35.73	99.70%	99.41%	98.66%	89.87%
**NEU2KO5**	600082196 (100%)	150599027 (25.14%)	599155157 (99.85%)	589931796 (98.31%)	598634698 (99.76%)	1040918 (0.17%)	5087474 (0.85%)	2411246 (0.40%)	29.99	99.68%	99.20%	96.14%	79.47%
**BAP1B3**	601438824 (100%)	97679377 (16.27%)	600365441 (99.82%)	588566622 (97.86%)	599758422 (99.72%)	1214038 (0.20%)	7041568 (1.17%)	3737350 (0.62%)	29.84	99.69%	99.26%	96.32%	78.79%
**BAP1B1**	670640902 (100%)	140193534 (20.94%)	669646637 (99.85%)	658814658 (98.24%)	669127946 (99.77%)	1037382 (0.15%)	6180214 (0.92%)	3033341 (0.45%)	33.42	99.69%	99.31%	97.04%	84.74%
**UV1KO2**	603725612 (100%)	85704640 (14.22%)	602646339 (99.82%)	591252720 (97.93%)	601923804 (99.70%)	1445070 (0.24%)	6956418 (1.15%)	4039588 (0.67%)	30.12	99.70%	99.37%	98.30%	81.50%
**UV1KO3**	603233460 (100%)	86188297 (14.32%)	602073080 (99.81%)	591280202 (98.02%)	601443394 (99.70%)	1259372 (0.21%)	6593212 (1.09%)	3532633 (0.59%)	30.12	99.68%	99.35%	98.35%	81.98%
**BAP2B1**	632734512 (100%)	114692629 (18.16%)	631528450 (99.81%)	619925402 (97.98%)	630836492 (99.70%)	1383916 (0.22%)	6617806 (1.05%)	3446314 (0.54%)	31.49	99.70%	99.29%	96.70%	82.45%
**BAP2KO2**	595801650 (100%)	111049345 (18.67%)	594895704 (99.85%)	583932428 (98.01%)	594336088 (99.75%)	1119232 (0.19%)	6744440 (1.13%)	3707693 (0.62%)	29.73	99.68%	99.34%	97.96%	78.44%
**NEU2B5**	608722484 (100%)	147516821 (24.27%)	607858152 (99.86%)	598378168 (98.30%)	607419412 (99.79%)	877480 (0.14%)	5122594 (0.84%)	2348691 (0.39%)	30.42	99.68%	99.24%	96.48%	80.65%
**NEU1KO2**	600403844 (100%)	159757186 (26.65%)	599509414 (99.85%)	590392444 (98.33%)	599075390 (99.78%)	868048 (0.14%)	4958910 (0.83%)	2303313 (0.38%)	29.99	99.65%	99.18%	96.28%	79.55%
**KONT3**	603275288 (100%)	149510209 (24.85%)	601668540 (99.73%)	590785006 (97.93%)	600853242 (99.60%)	1630596 (0.27%)	6413720 (1.06%)	3470591 (0.58%)	29.74	99.70%	99.17%	95.75%	77.10%
**A2NT3**	619684754 (100%)	162793693 (26.34%)	617983723 (99.73%)	604960074 (97.62%)	617073682 (99.58%)	1820082 (0.29%)	8149284 (1.32%)	4962993 (0.80%)	30.59	99.71%	99.22%	96.16%	79.39%
**BAP1A3**	607659472 (100%)	113665763 (18.75%)	606246650 (99.77%)	595904040 (98.07%)	605219976 (99.60%)	2053348 (0.34%)	5663482 (0.93%)	2817275 (0.46%)	30.15	99.68%	99.21%	96.10%	79.64%
**NEU1B4**	679478658 (100%)	150374984 (22.16%)	678468537 (99.85%)	667696042 (98.27%)	677942716 (99.77%)	1051642 (0.15%)	5846012 (0.86%)	2725941 (0.40%)	33.95	99.70%	99.34%	97.39%	86.70%
**NEU1B6**	646493458 (100%)	161526481 (25.03%)	645310387 (99.82%)	635053614 (98.23%)	644664466 (99.72%)	1291842 (0.20%)	5510148 (0.85%)	2531277 (0.39%)	32.26	99.69%	99.29%	96.96%	83.95%
**BAP1A8**	600783348 (100%)	119319400 (19.89%)	599960573 (99.86%)	590581720 (98.30%)	599493430 (99.79%)	934286 (0.16%)	5085444 (0.85%)	2577221 (0.43%)	30.00	99.68%	99.21%	96.08%	78.63%
**BAP2A7**	626522628 (100%)	111862616 (17.91%)	624666777 (99.70%)	613114382 (97.86%)	623267698 (99.48%)	2798158 (0.45%)	5989800 (0.96%)	2931570 (0.47%)	31.09	99.71%	99.28%	96.45%	81.32%
**B4NT3**	599598080 (100%)	147097715 (24.60%)	597989695 (99.73%)	587704850 (98.02%)	597090902 (99.58%)	1797586 (0.30%)	5449238 (0.91%)	2560871 (0.43%)	29.60	99.71%	99.23%	96.06%	77.71%
**UV3UV1**	874725228 (100%)	180271567 (20.64%)	873417878 (99.85%)	857101062 (97.99%)	872802532 (99.78%)	1230692 (0.14%)	9814196 (1.12%)	5618273 (0.64%)	43.56	99.73%	99.48%	99.03%	96.05%
**BAP1KO3**	599221854 (100%)	128024509 (21.40%)	598345971 (99.85%)	588820034 (98.26%)	597851464 (99.77%)	989014 (0.17%)	5257656 (0.88%)	2462153 (0.41%)	29.95	99.69%	99.35%	98.08%	79.82%
**NEU2A6**	765471482 (100%)	238411435 (31.20%)	764113326 (99.82%)	751850688 (98.22%)	763498092 (99.74%)	1230468 (0.16%)	6593520 (0.86%)	3061582 (0.40%)	38.15	99.71%	99.40%	98.02%	89.83%
**NEU1KO1**	596889342 (100%)	167734924 (28.14%)	596074926 (99.86%)	586254294 (98.22%)	595563058 (99.78%)	1023736 (0.17%)	5656810 (0.95%)	2840630 (0.48%)	29.76	99.65%	99.16%	96.12%	78.79%
**NEU2A5**	600898310 (100%)	170130421 (28.35%)	600041522 (99.86%)	591462492 (98.43%)	599626762 (99.79%)	829520 (0.14%)	4498740 (0.75%)	2106091 (0.35%)	30.00	99.65%	99.16%	96.04%	77.61%
**UV4UV2**	603024112 (100%)	148230447 (24.62%)	602100839 (99.85%)	593013412 (98.34%)	601567902 (99.76%)	1065874 (0.18%)	4737322 (0.79%)	2145262 (0.36%)	30.18	99.68%	99.33%	97.72%	79.59%

Appendix Table S6.

Statistics on mapping, coverage and sequencing depth for samples.

Study link	Cancer type	Cell line/ sample	AA change
https://doi.org/10.1371/journal.pcbi.1005725	Melanoma	Mel15	SF
https://doi.org/10.1371/journal.pcbi.1005725	Melanoma	Mel15	PL
https://doi.org/10.1371/journal.pcbi.1005725	Melanoma	Mel15	PL
https://doi.org/10.1371/journal.pcbi.1005725	Melanoma	Mel15	TI
https://doi.org/10.1371/journal.pcbi.1005725	Melanoma	Mel15	PL
https://doi.org/10.1371/journal.pcbi.1005725	Melanoma	Mel15	MI
https://doi.org/10.1371/journal.pcbi.1005725	Melanoma	Mel15	EK
https://doi.org/10.1371/journal.pcbi.1005725	Melanoma	Mel15	SF
https://doi.org/10.1371/journal.pcbi.1005725	Melanoma	Mel8	PL
https://doi.org/10.1371/journal.pcbi.1005725	Melanoma	Mel5	EK
https://doi.org/10.1371/journal.pcbi.1005725	Melanoma	Mel5	QR
https://doi.org/10.18632/oncotarget.6960	Melanoma	pat_12T	PS
https://doi.org/10.18632/oncotarget.6960	Melanoma	pat_12T	SL
https://doi.org/10.1021/acs.analchem.8b02420	Colon carcinoma	HCT_116	VI
https://doi.org/10.1021/acs.analchem.8b02420	Colon carcinoma	HCT_116	RW
https://doi.org/10.1021/acs.analchem.8b02420	Colon carcinoma	HCT_116	VA
https://doi.org/10.1021/acs.analchem.8b02420	Colon carcinoma	HCT_116	GV
https://doi.org/10.1021/acs.analchem.8b02420	Colon carcinoma	HCT_116	RH
https://doi.org/10.1021/acs.analchem.8b02420	Colon carcinoma	HCT_116	SP
https://doi.org/10.1021/acs.analchem.8b02420	Colon carcinoma	HCT_116	AS
https://doi.org/10.1021/acs.analchem.8b02420	Colon carcinoma	HCT_116	CR
https://doi.org/10.1021/acs.analchem.8b02420	Colon carcinoma	HCT_116	TP
https://doi.org/10.1021/acs.analchem.8b02420	Colon carcinoma	HCT_116	ML
https://doi.org/10.1007/s00262-019-02358-0	Melanoma	A375	VL
https://doi.org/10.1007/s00262-016-1897-3	Melanoma	MEWO	HY
https://doi.org/10.1186/s40425-019-0769-8	Colon carcinoma	CRC-01	TR
https://doi.org/10.1186/s40425-019-0769-8	Colon carcinoma	CRC-01	KT
https://doi.org/10.1186/s40425-019-0769-8	Colon carcinoma	CRC-04	SN
https://doi.org/10.1172/jci.insight.146356	Colon carcinoma	CRC-111	AT
https://doi.org/10.1038/s41467-025-62647-4	Melanoma	MI1	CR
https://doi.org/10.1038/s41467-025-62647-4	Melanoma	MI2	DN
https://doi.org/10.1016/j.cell.2017.09.050	Melanoma	A375	AT
https://doi.org/10.1016/j.cell.2017.09.050	Melanoma	A375	IT
https://doi.org/10.1016/j.cell.2017.09.050	Melanoma	A375	VI
https://doi.org/10.1016/j.cell.2017.09.050	Melanoma	A375	NS
https://doi.org/10.1016/j.cell.2017.09.050	Ovarian cancer	A2780	MV
https://doi.org/10.1016/j.cell.2017.09.050	Ovarian cancer	A2780	MT
https://doi.org/10.1016/j.cell.2017.09.050	Ovarian cancer	A2780	LF
https://doi.org/10.1016/j.cell.2017.09.050	Ovarian cancer	OV90	PA
https://doi.org/10.1016/j.cell.2017.09.050	Ovarian cancer	OV90	VF
https://doi.org/10.1016/j.cell.2017.09.050	Cervical cancer	HeLa	PL
https://doi.org/10.1016/j.cell.2017.09.050	Ovarian cancer	SKOV3	LV
https://doi.org/10.1016/j.cell.2017.09.050	Ovarian cancer	SKOV3	YH
https://doi.org/10.1016/j.cell.2017.09.050	Ovarian cancer	SKOV3	ED
https://doi.org/10.1016/j.cell.2017.09.050	Ovarian cancer	SKOV3	PQ
https://doi.org/10.1016/j.cell.2017.09.050	Ovarian cancer	SKOV3	RH
https://doi.org/10.1111/imm.13578	Esophageal cc.	EN-454-11	GR

Appendix Table S7.

List of amino acid substitutions identified in 17 different samples of immunopeptidomics studies.
