# Supplementary material for: Five dominant amino acid substitution signatures shape tumour immunity
Source: Mol Syst Biol. 2026 Jan 28;22(5):766–86. doi: 10.1038/s44320-026-00193-x (PMC13144524; doi:10.1038/s44320-026-00193-x)
Supplement: Supplementary file 2 — Table EV1 [file 44320_2026_193_MOESM2_ESM.pdf]

Table EV1. Characterization of AASs. The table summarizes the five distinct AAS signatures (AAS1 to AAS5) based on specific nucleotide mutations, single base substitutions (SBS), associated mutagens or biological processes, gene knockouts (KO), amino acid substitutions, and cancer types. Of note, nucleotide substitutions are represented relative to the pyrimidine bases to avoid redundancy and simplify interpretation (see Figure 2A for details). ROS: reactive oxygen species. MMR: mismatch repair. HR: homologous recombination. ENU: N-ethyl-N-nitrosourea.

	AAS1	AAS2	AAS3	AAS4	AAS5
Nucleotide mutations	5' C/G **C > A** A/C/T 3'	5' C/T **C > T** C/T 3'	5' A/C/G **C > T** G 3'	5' C **T > C** G<br>T **T > G** T 3'	5' T **C > G** A/T 3'
SBS	4, 8, 18, 24, 29, 35, 36	2, 7a, 7b, 11, 30	1, 6, 15	3, 5, 9, 12, 25, 26, 37, 40, 41<br>44, 46	13
Associated mutagens/ processes	Cigarette smoke<br>Aflatoxin A1<br>Cisplatin, ROS (KBrO3)	UV light<br>APOBEC3<br>Temozolomide	MMR deficiency<br>Clock-like sign.	MMR deficiency<br>HR deficiency<br>Alkilating agents (ENU)<br>Glycidamide<br>Clock-like sign.	APOBEC3
Gene KO	OGG1<br>MUTYH	NTHL1<br>UNG	PMS1	MLH1, MSH2, PMS2, MSH6<br>EXO1, RNF168	-
AA substitutions<br>Hydrophobic, polar, negative, positive, special	Gly > Val<br>Ala > Ser	Glu > Lys, Pro > Ser<br>Asp > Asn, Gly > Glu<br>Ser > Phe, Pro > Leu	Arg > His, Arg > Cys<br>Arg > Gln, Arg > Trp<br>Ala > Val, Ala > Thr	Heterogeneous	Glu > Lys<br>Glu > Gln
Cancer types	LUAD<br>LUSC<br>LIHC	SKCM	COAD, READ<br>PAAD, STAD<br>UCEC, PRAD	KIRC<br>KIRP<br>LIHC<br>THCA	BLCA<br>CESC
