# Supplementary material for: Five dominant amino acid substitution signatures shape tumour immunity
Source: Mol Syst Biol. 2026 Jan 28;22(5):766–86. doi: 10.1038/s44320-026-00193-x (PMC13144524; doi:10.1038/s44320-026-00193-x)

LP_ 612
DAY 7

ID	RACE	AGE	GENDER	HLA A	HLA B
**LP_612**	Caucasian	36	Male	A*01:01	B*07:02
				A*03:01	B*40:01

CFSE negativecells | CTRL (CFSE positive) | BEAD | HLA KO_NEU | HLA A*03:01_NEU | HLA B*07:02_NEU

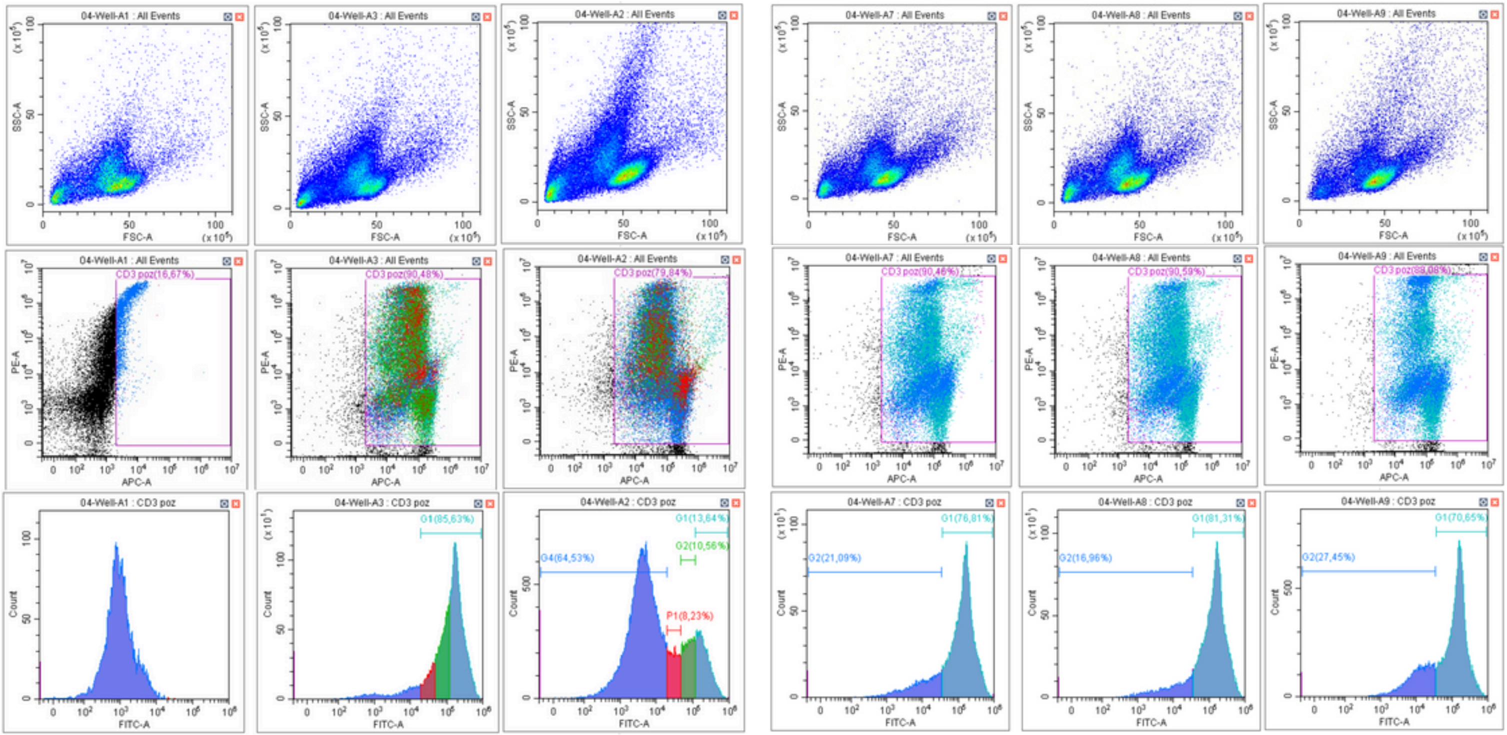

LP_620
DAY 7

ID	RACE	AGE	GENDER	HLA A	HLA B
**LP_620**	Caucasian	24	Female	A*02:01<br>A*03:01	B*07:02<br>B*13:02

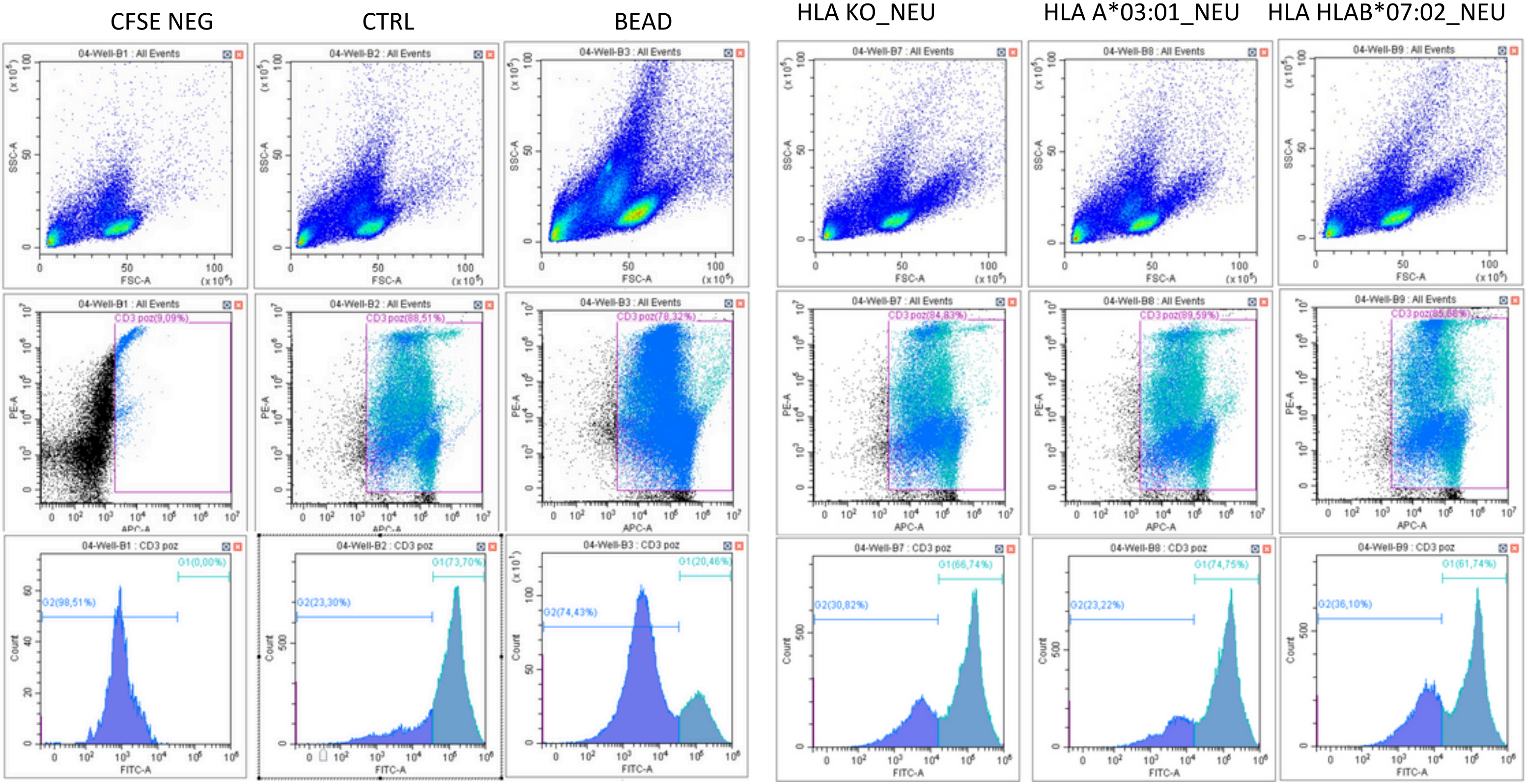

LP_585
DAY 7

ID	RACE	AGE	GENDER	HLA A	HLA B
**LP_585**	Caucasian	48	Male	A*01:01<br>A*02:01	B*07:02<br>B*40:01

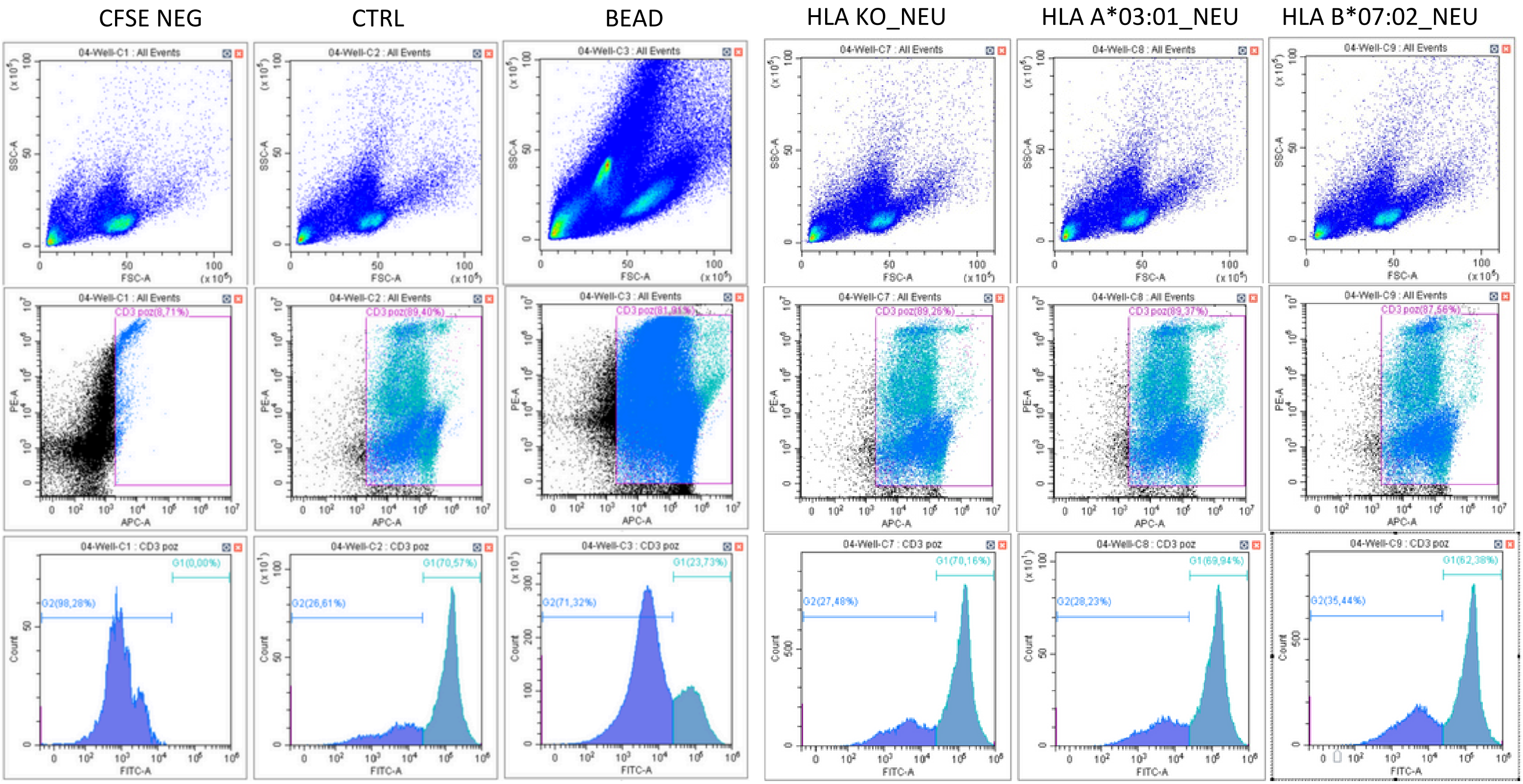

LP_609
DAY 7

ID	RACE	AGE	GENDER	HLA A	HLA B
**LP_609**	Caucasian	46	Female	A*01:01<br>A*01:01	B*08:01<br>B*07:02

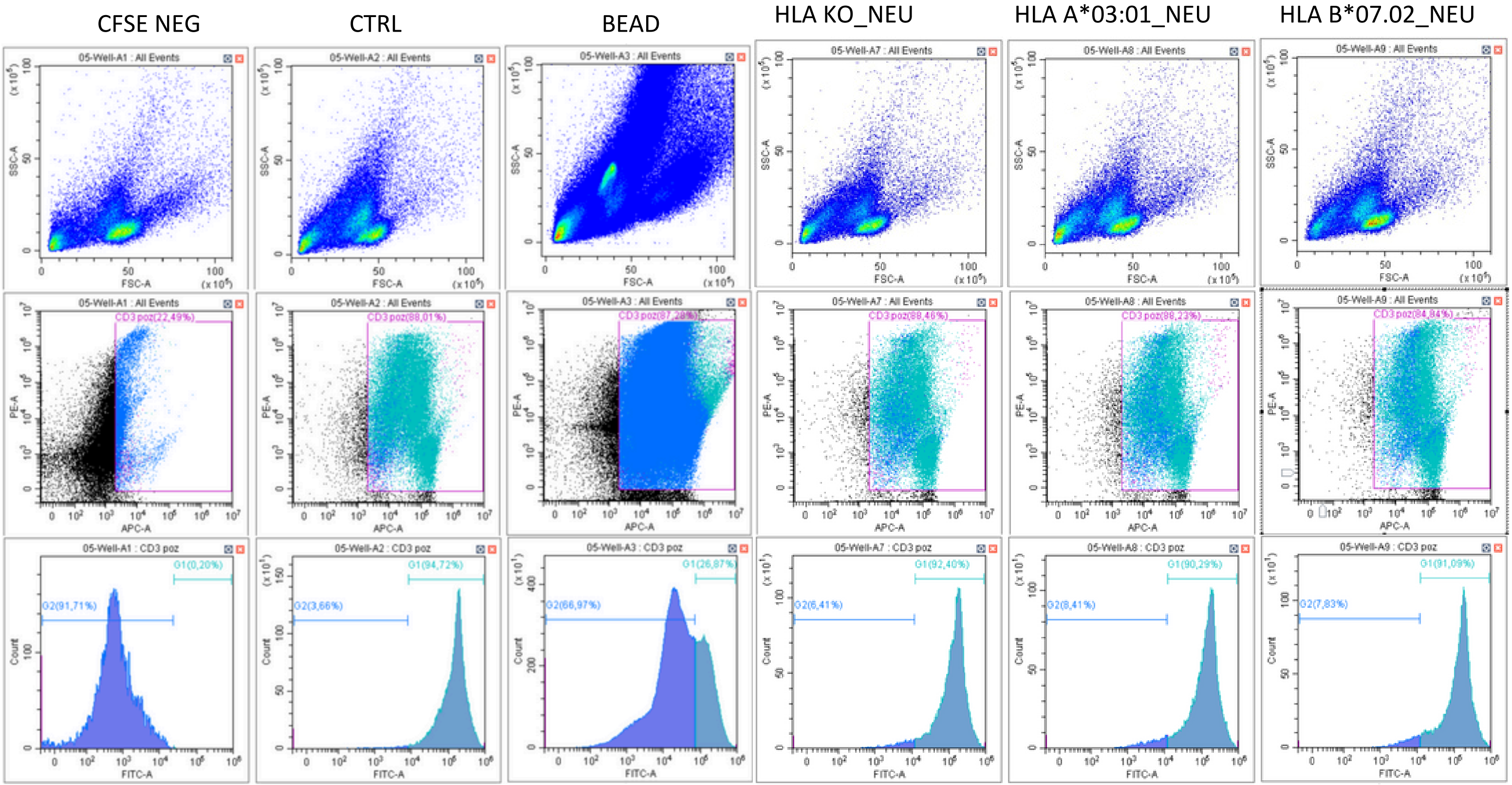

LP_591

DAY 7

ID	RACE	AGE	GENDER	HLA A	HLA B
**LP_591**	Caucasian	27	Male	A*02:01	B*15:03
				A*03:01	B*40:01

CFSE NEG	CTRL	BEAD	HLA KO_NEU	HLA A*03:01_NEU.	HLA B*07:02_NEU

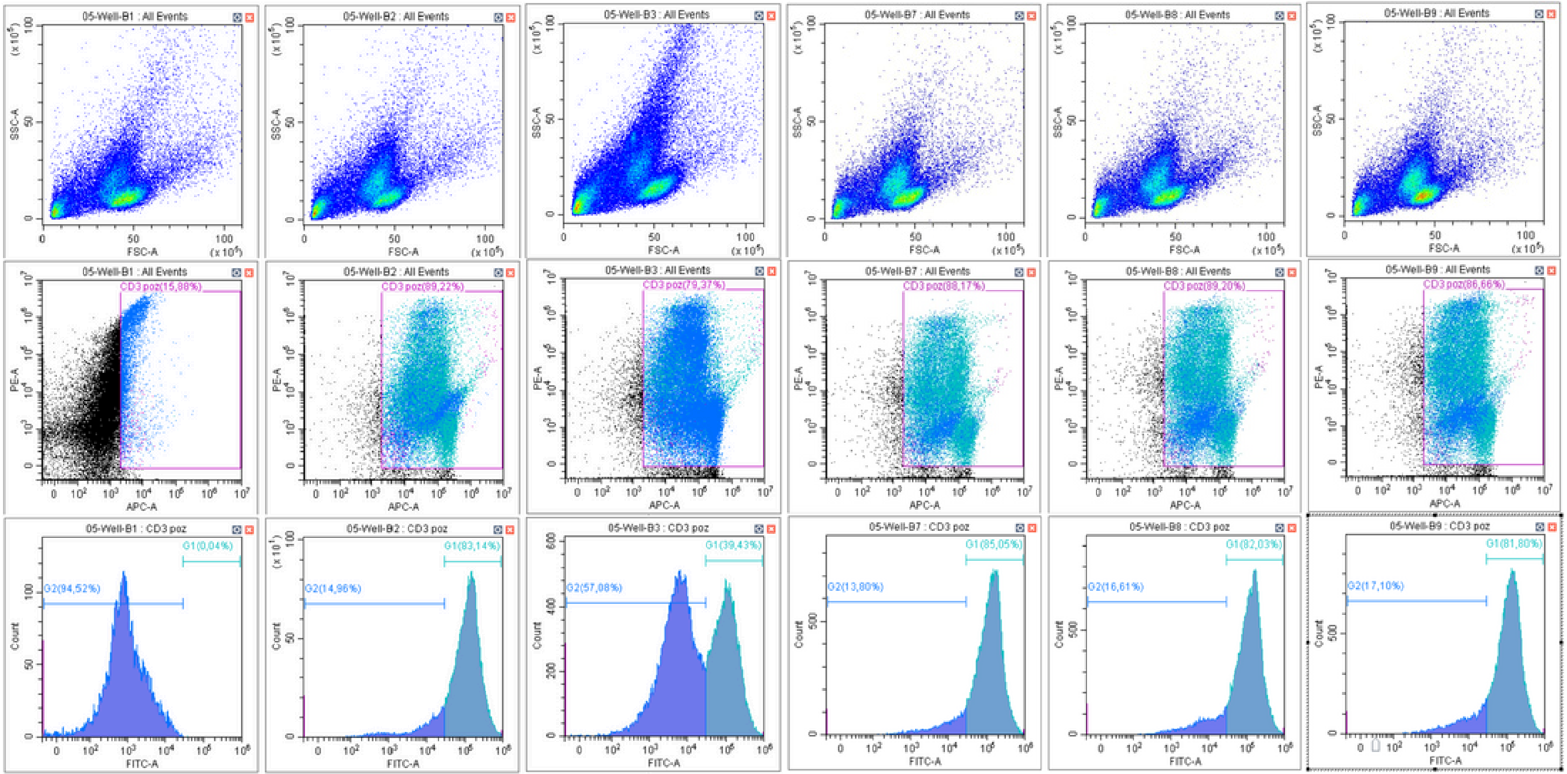

LP_474
DAY 7

ID	RACE	AGE	GENDER	HLA A	HLA B
**LP_474**	Caucasian	32	Male	A*03:01<br>A*23:01	B*35:01<br>B*49:01

CFSE NEG | CTRL | BEAD | HLA KO_NEU | HLA A*03:01_NEU | HLA B*07:02_NEU

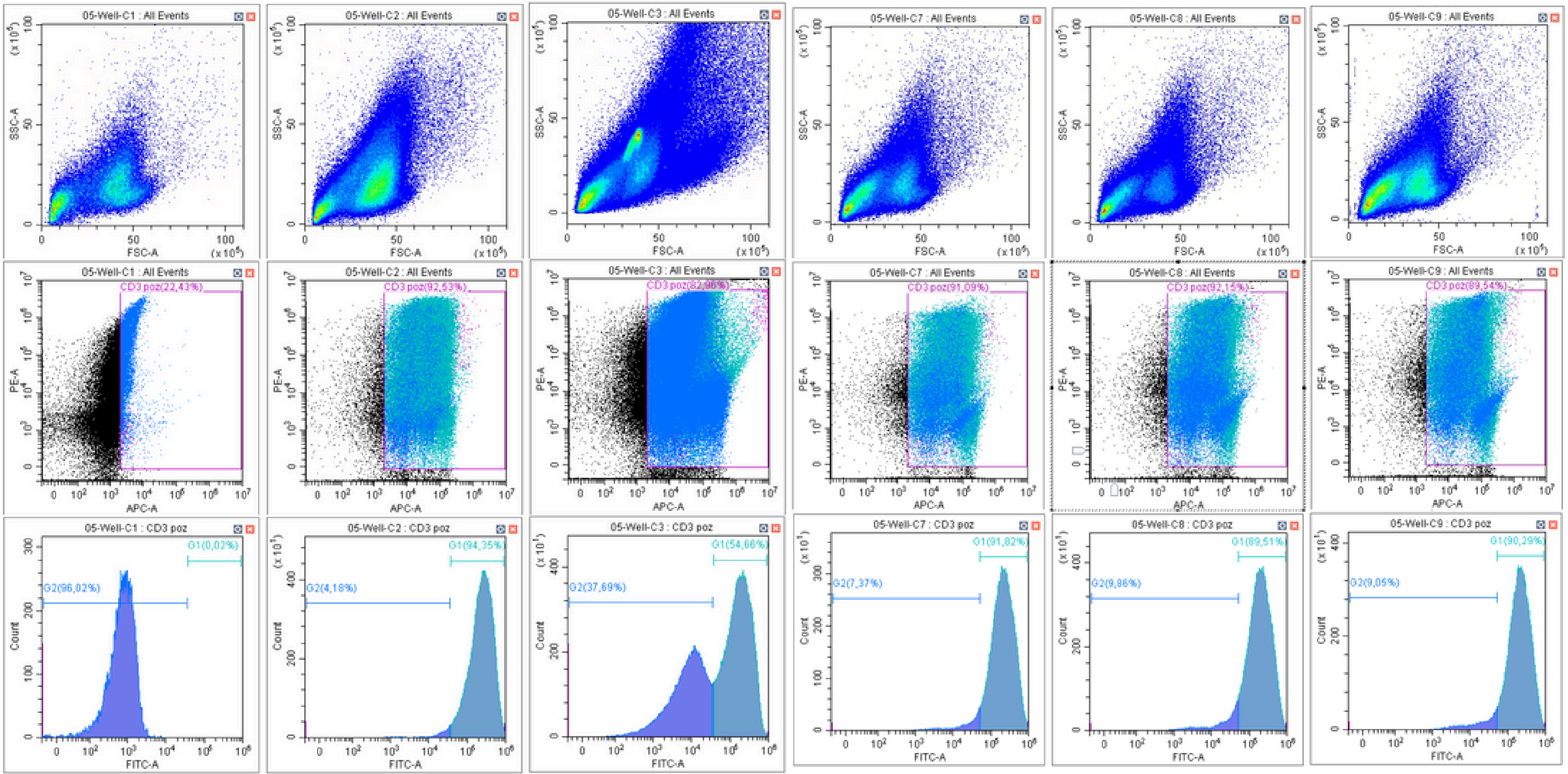

LP_ 470
DAY 7

ID	RACE	AGE	GENDER	HLA A	HLA B
**LP_470**	Hispanic	43	Male	A*03:01	B*07:02
				A*03:01	B*58:01

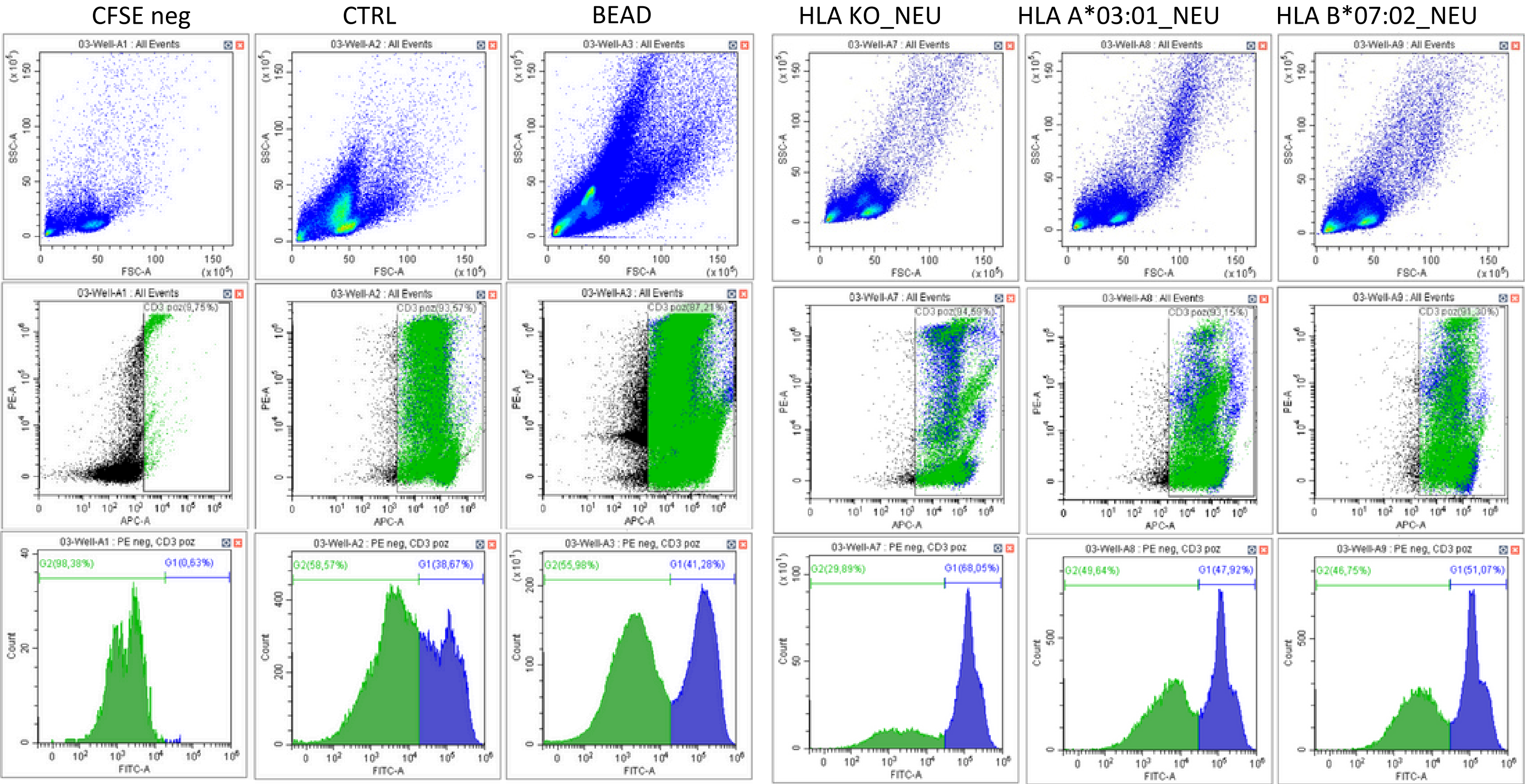

LP_ 607
DAY 7

ID	RACE	AGE	GENDER	HLA A	HLA B
**LP_607**	African/American	29	Female	A*02:01<br>A*03:01	B*07:02<br>B*51:01

CFSE neg | CTRL | BEAD | HLA KO_NEU | HLA A*03:01_NEU | HLA B*07:02_NEU

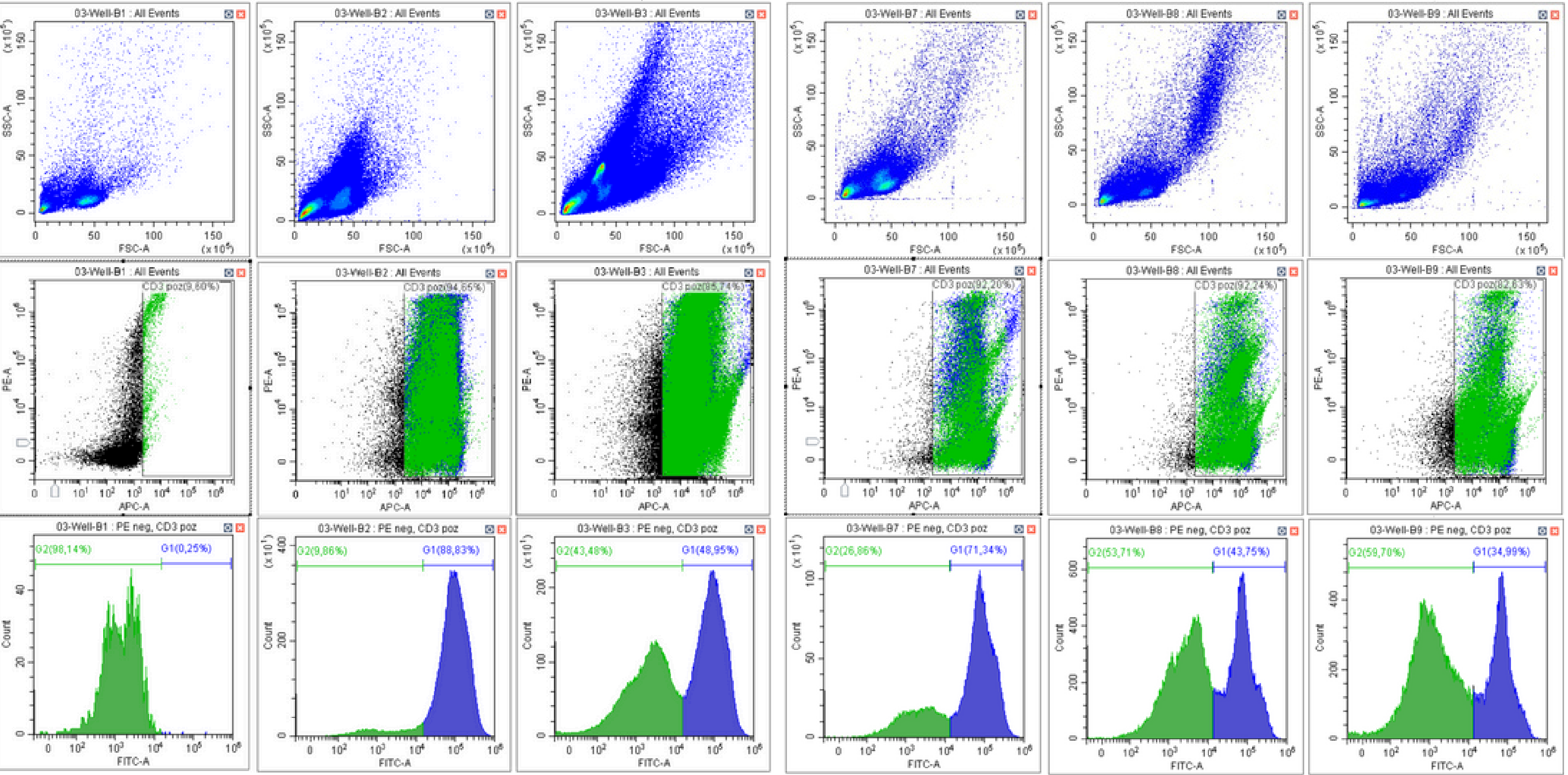

LP_ 464
DAY 7

ID	RACE	AGE	GENDER	HLA A	HLA B
**LP_464**	Caucasian	40	Female	A*01:01<br>A*11:01	B*07:02<br>B*08:01

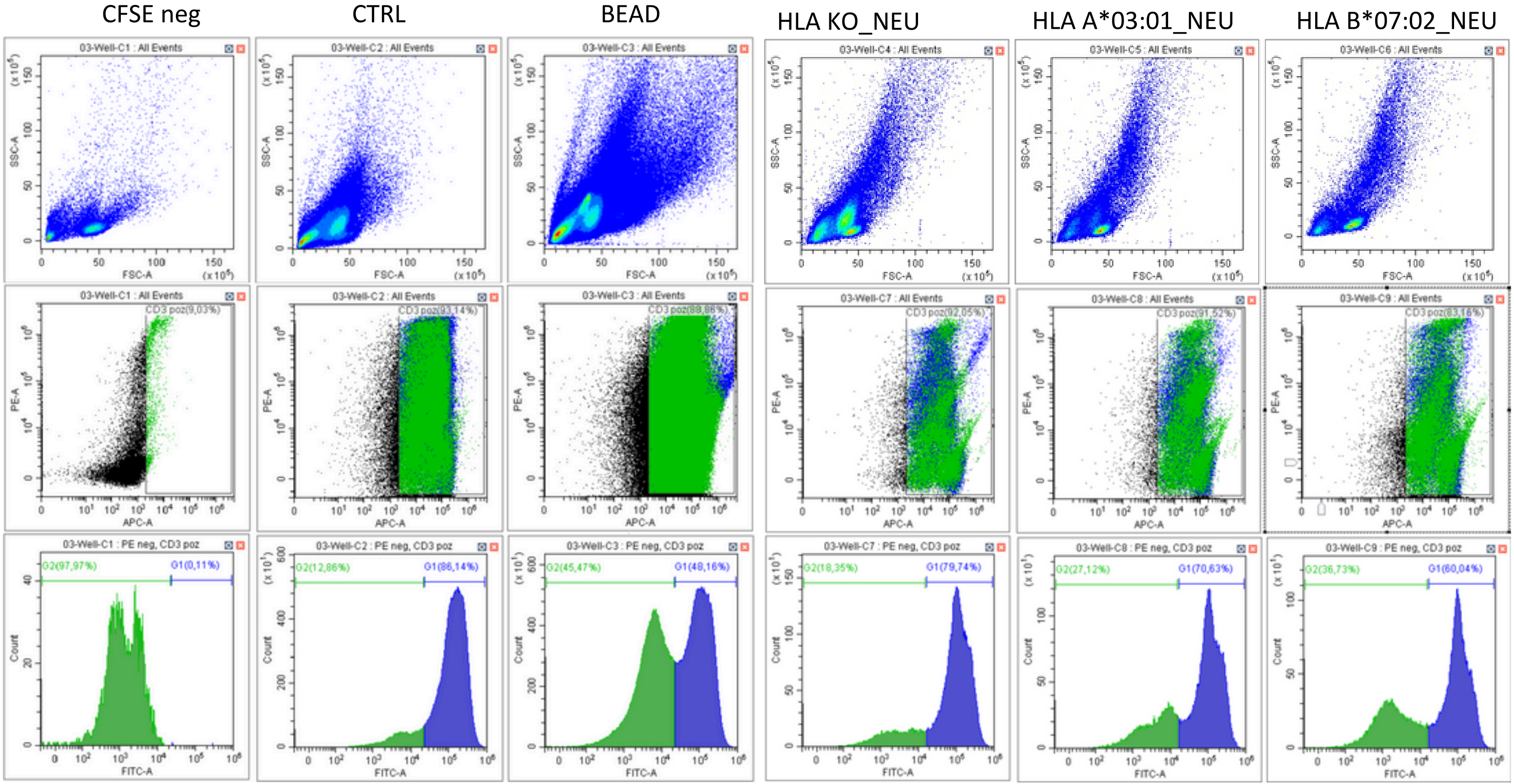

LP_ 533
DAY 7

ID	RACE	AGE	GENDER	HLA A	HLA B
**LP_533**	Hispanic	40	Male	A*02:02<br>A*30:02	B*07:02<br>B*15:10

CFSE neg | CTRL | BEAD | HLA KO_NEU | HLA A*03:01_NEU | HLA B*07:02_NEU

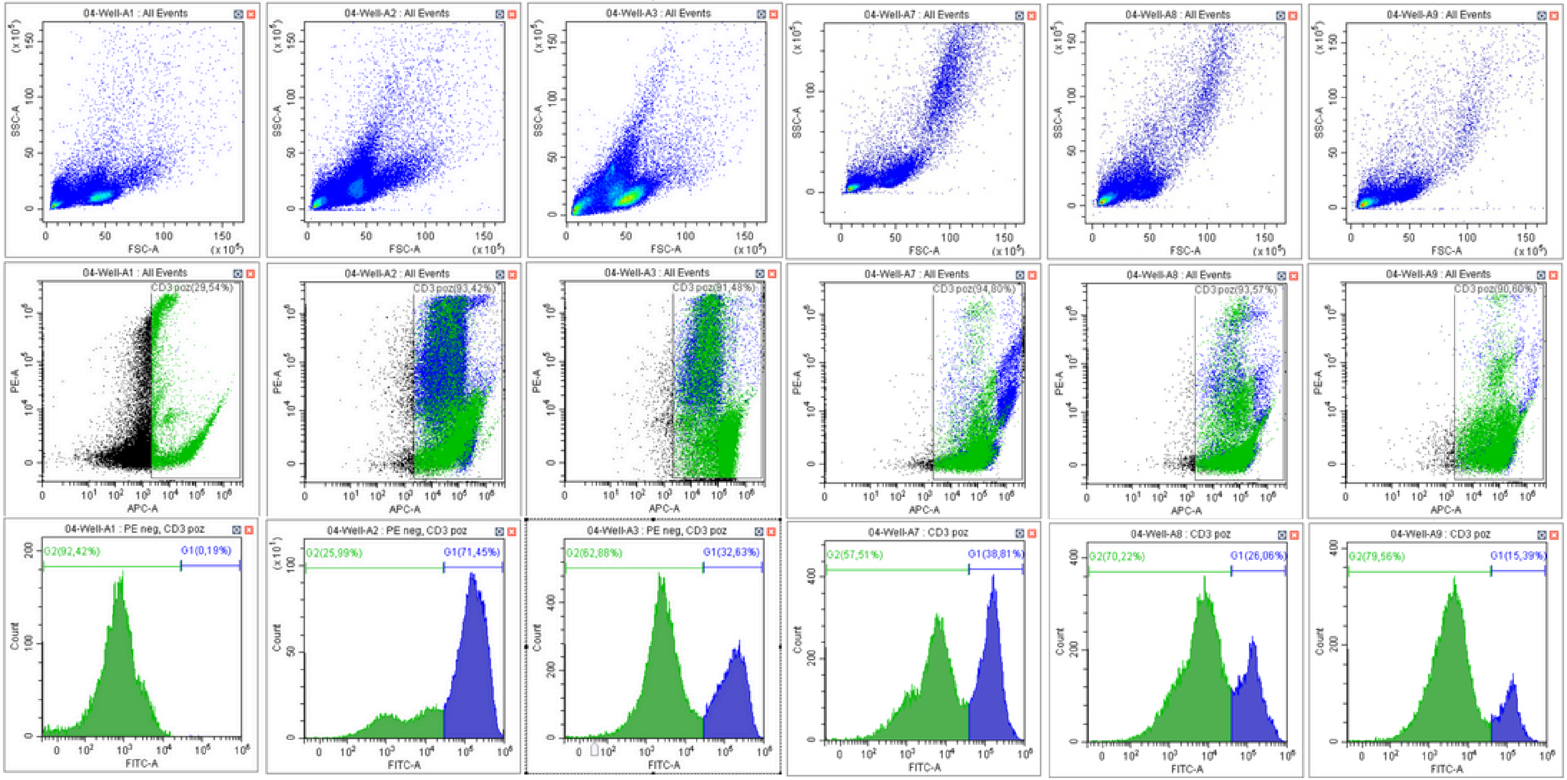

LP_ 363
DAY 7

ID	RACE	AGE	GENDER	HLA A	HLA B
**LP_363**	Hispanic/Latino	28	Female	A*03:01<br>A*24:02	B*08:01<br>B*35:01

CFSE neg | CTRL | BEAD | HLA KO_NEU | HLA A*03:01_NEU | HLA B*07:02_NEU

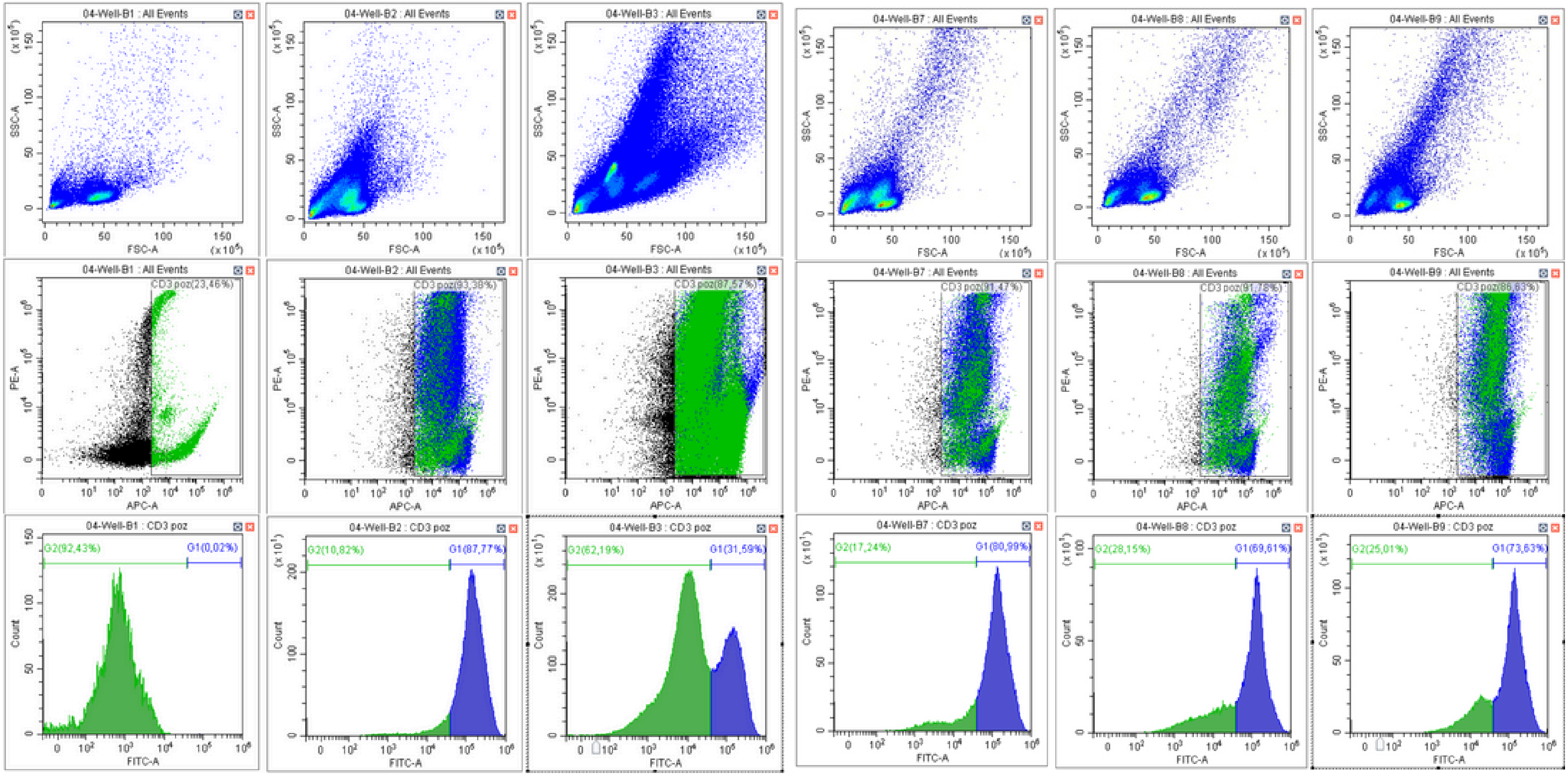

LP_ 220
DAY 7

ID	RACE	AGE	GENDER	HLA A	HLA B
**LP_220**	African/American	31	Male	A*03:01<br>A*29:02	B*15:17<br>B*44:03

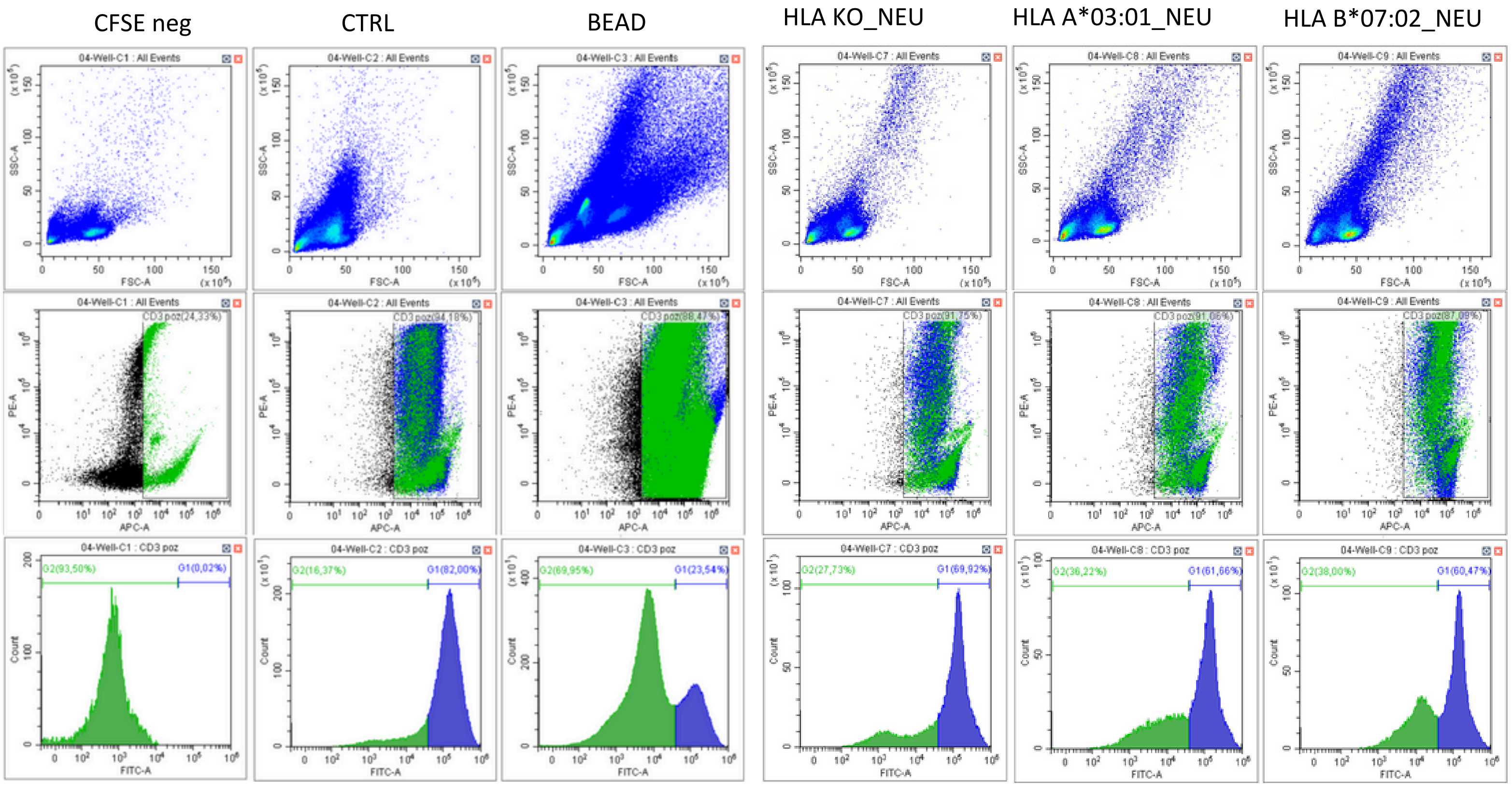

Supplement: Supplementary file 9 — Source data Fig. 7 [file 44320_2026_193_MOESM9_ESM.zip › Figure_7/D/flow_cytometry_data.pdf]
